# Association of accelerometer-measured physical activity and the risk of incident cardiovascular diseases within the AHA cardiovascular-kidney-metabolic syndrome 0–4 framework: a prospective cohort of the UK Biobank

**DOI:** 10.3389/fendo.2026.1844167

**Published:** 2026-07-31

**Authors:** Jun-Qiao An, Xue He, Jing-Yi Ding, Yan Wu, Xiang-Wei Luan, Zhao-Zong Xu, Tian-Tian Guo, Qing-Yong He

**Affiliations:** 1 Beijing University of Chinese Medicine, Beijing, China; 2 Guang'anmen Hospital, China Academy of Chinese Medical Sciences, Beijing, China

**Keywords:** dose-response relationship, isotemporal substation models, lifestyle medicine, multimorbidity, wearable electronic devices

## Abstract

**Background:**

Cardiovascular-kidney-metabolic (CKM) syndrome spans stages 0–4, with stage 4 denoting clinical cardiovascular disease (CVD). While individuals in stages 0–3 face elevated incident CVD risk, how different durations of physical activity (PA) associate with this risk remains unclear.

**Methods:**

This prospective UK Biobank cohort included 88,281 participants aged 37–73 in CKM syndrome stages 0–3, excluding those with baseline CVD. Overall and stage-specific analyzes were performed to evaluate the associations between PA and incident CVD risk. PA was categorized into light PA (LPA) and moderate-to-vigorous PA (MVPA), then divided into quartiles by weekly duration. Multivariable Cox models were used to estimate hazard ratios. An isochronous substitution model using component analysis was used to assess the associated risk differences of replacing LPA with MVPA.

**Results:**

During a median follow-up of 7.29 years, 7,514 new CVD cases occurred among 88,281 individuals with CKM syndrome stages 0-3. LPA was associated with a lower CVD risk most markedly in stage 2 individuals, with the highest quartile showing a 18% lower risk versus the lowest (HR = 0.82; 95% CI 0.75-0.90). MVPA was associated with a lower CVD risk in stages 1 and 2, with the highest quartile associated with a 42% (HR = 0.58; 95% CI 0.50-0.66) and 33% (HR = 0.67; 95% CI 0.61-0.73) lower risk, respectively. Overall, LPA and MVPA exhibited nonlinear L-shaped relationships across stages 0–3, with overall inflection points at 2,357 (95% CI: 2335-2379) min/week for LPA and 213 (95% CI: 207-219) min/week for MVPA. Specifically, MVPA in stage 3 population exhibited a U-shaped CVD risk curve, increasing beyond 460 (95%CI: 449-472) min/week. In the general population at stages 0-3, when LPA exceeded 2,551 min/week, third-quartile MVPA was associated with a similarly lower risk as the fourth quartile. Substituting MVPA for LPA was associated with further lower risk in stages 1 and 2, while these inverse associations plateaued or declined after reaching 350 min/week in stage 0 and 300 min/week in stage 3, respectively.

**Conclusion:**

Associations between physical activity and incident CVD differ across CKM stages 0–3. These stage-specific differences for LPA and MVPA warrant further validation to inform future guidelines.

## 1 Introduction

In the general population, low-intensity physical activity (LPA) constitutes the major portion of daily physical activity, followed by moderate-to-vigorous physical activity (MVPA). The World Health Organization (WHO) guidelines recommend that LPA and MVPA are lower-risk physical activities for individuals with cardiovascular risk factors and are recommended as a preventive intervention (1). The guidelines specify that adults should engage in 150 to 300 minutes of moderate-intensity physical activity (e.g., brisk walking, swimming) weekly. Constituting a major component of daily energy expenditure, LPA is closely associated with daily living activities and any form of routine movement (e.g., walking, climbing stairs) (2). However, its significant role has long been underappreciated (3). Therefore, comprehensively grasping the duration and combination of LPA and MVPA is key to optimizing individual physical activity patterns, particularly among those with cardiovascular risk factors (4, 5).

In 2023, the American Heart Association (AHA) introduced the cardiovascular-kidney-metabolic (CKM) syndrome, proposing a more refined 5-stage risk stratification for CVD prevention (6): from stage 0 with no CKM risk factors, to stage 1 characterized by overweight, obesity or prediabetes, then to stage 2 where metabolic diseases (such as hypertension, hypertriglyceridemia, diabetes) develop. Stage 3 reflects subclinical CVD or high-risk CKD on top of stages 1–2 risk factors, whereas stage 4 mainly covers clinical CVD. A nationwide survey conducted between 2011 and 2020 revealed that only 10.6% of Americans met the criteria for CKM syndrome stage 0, while 25.9%, 49.0%, 5.4% and 9.2% of the population met the criteria for stages 1, 2, 3 and 4, respectively (7, 8). While the AHA framework comprehensively spans stages 0 through 4, our study focuses specifically on the preclinical-to-subclinical segment of CKM (stages 0–3) prior to clinical CVD (9). Global estimates from the 2026 Heart Disease and Stroke Statistics indicated that CVD affected approximately 610 million individuals and caused close to 20 million deaths (10). Therefore, to address this substantial disease burden, translational integrative lifestyle medicine should be widely implemented. This comprehensive strategy must include personalized exercise recommendations adapted to the distinct characteristics across all CKM syndrome stages to effectively address disease prevention and progression (11).

While the importance of stage-specific lifestyle management for CKM syndrome is increasingly recognized, accelerometer-based data detailing optimal LPA and MVPA duration and combination associated with the lowest cardiovascular risk across CKM syndrome stages 0–3 is still lacking. Our study aims to utilize objectively measured PA data from accelerometers in the large-scale UK Biobank cohort to explore the associations between various LPA and MVPA patterns and the risk of incident CVD in individuals across stages 0–3 of CKM syndrome. The research findings will provide scientific evidence to generate hypotheses and inform future studies aimed at refining stage-specific public health recommendations targeting this high-risk population of CVD.

## 2 Methods

### 2.1 Data source and study population

**Figure 1:**
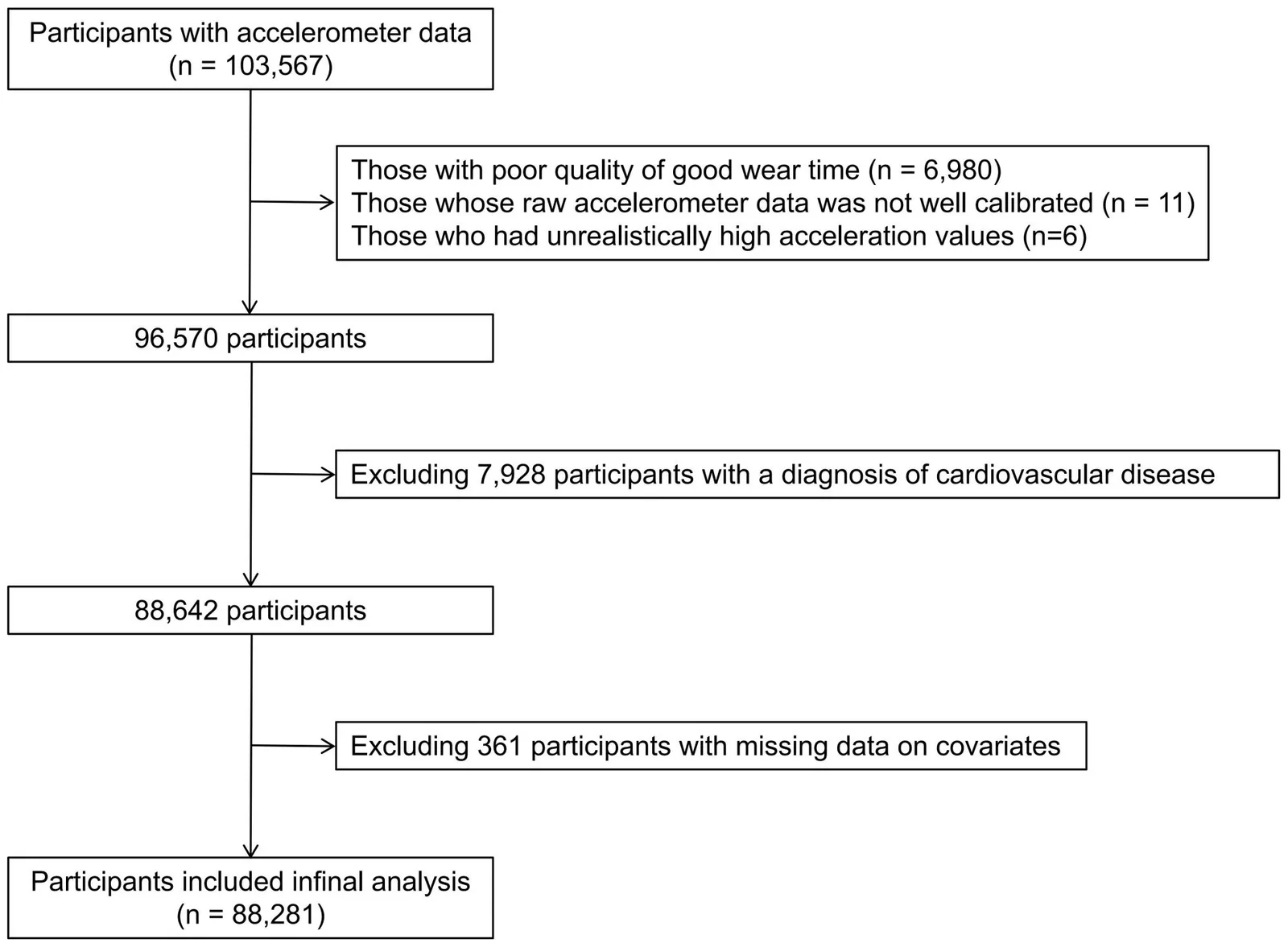
Flowchart of inclusion and exclusion of study participants.

The data of this study were derived from the UK Biobank cohort. This cohort recruited 502,629 participants, with the 37–73-year age range explicitly reflecting the established baseline recruitment eligibility criteria for the study (12). As a significant sub-study of UK Biobank, a subset of 103,567 participants was invited to wear a wrist-worn accelerometer for seven consecutive days to provide objective physical activity(PA) measurements between 2013 and 2015. The sub-study participants were followed up for incident health outcomes from the date of their PA measurement through November 30, 2022. We sequentially excluded the following individuals from the 103,567 participants wearing accelerometers: insufficient valid wear time (n=6,980), poor accelerometer data calibration (n=11), and outlier measurements (n=6). After further excluding participants diagnosed with CVD at baseline (n=7,928) and those with missing covariate data (n=361), a final cohort of 88,281 participants was included (**Figure 1**).

The UK Biobank was approved by the UK National Health Service Research Ethics Committee (approval number: 21/NW/0157), and all participants provided written informed consent at the time of recruitment for the use of their data in future research. The present study is a secondary analysis of the UK Biobank data, conducted under the approved UK Biobank application ID 906,426. All analyzes for this study were performed in accordance with relevant guidelines and regulations and were carried out using de-identified data to ensure participant confidentiality.

### 2.2 Exposures

Participants wore the Axivity AX3 triaxial accelerometer (Axivity Ltd, Newcastle, UK) on their dominant wrist continuously for 7 days. A validated random forest machine learning algorithm was employed to classify accelerometer data into mutually exclusive behaviors, including sedentary behavior, LPA, MVPA, and sleep. The exposure variables for this study were directly extracted from the “Derived accelerometry” category of the UK Biobank database, specifically utilizing the “Light - Overall average” and “Moderate-Vigorous - Overall average” data fields. The data preprocessing was strictly based on the standard UK Biobank accelerometry pipeline (13). This embedded pipeline automatically handles sensor calibration, identifies and imputes non-wear periods (typically defined as consecutive stationary episodes lasting ≥60 minutes), and establishes valid-wear thresholds (14). Participants were considered to have valid wear time if they provided at least 72 hours of data, including data in each 24-hour cycle, in accordance with the established UK Biobank technical processing criteria. These values, representing the average proportion of time spent in each activity intensity, were used to estimate the total weekly duration of LPA and MVPA for each participant. The grouping of LPA and MVPA into quartiles was performed at two levels. First, overall quartiles were calculated based on the entire study population (CKM stages 0–3 combined) to provide a comprehensive overview of PA patterns for this whole population. Second, to formulate more refined guidance, stage-specific quartiles were calculated separately within each individual CKM stage population.

### 2.3 Outcomes

The primary outcome of this study was new-onset cardiovascular disease (CVD) events, defined according to the International Classification of Diseases (ICD-10) criteria and encompassing codes I20-I25, I60-I64, and I50. Follow-up began at baseline and ended at the first occurrence of a CVD event, death, or the last follow-up date (whichever occurred first). To minimize potential reverse causality effects, we analyzed the models by excluding all participants who experienced a CVD event or died within the first 2 years of follow-up. In these analyzes, both the early incident cases and their corresponding person-years contributed during this initial 2-year window were removed. A similar approach was applied in a sensitivity analysis using a 5-year exclusion period.

### 2.4 CKM syndrome stages

According to the American Heart Association (AHA) guidelines, CKM syndrome is officially defined as a five-stage framework ranging from Stage 0 (no risk factors) to Stage 4 (established cardiovascular disease) (6). Because this study focused on incident events and excluded all individuals with baseline clinical CVD, our primary analysis was restricted to Stages 0 through 3. Each participant was hierarchically assigned to the highest applicable CKM stage based on their most advanced clinical condition or risk factor at baseline. Stage 0 (No CKM Risk Factors): Refers to individuals with a body mass index (BMI) between 18.5 and 24.9, a waist circumference <88 cm for females or <102 cm for men, and no other risk factors. Stage 1 (Excess/Dysfunctional Adiposity): Characterized by meeting at least one of the following criteria: (i) BMI ≥25 kg/m^2^; (ii) waist circumference ≥88 cm in females or ≥102 cm in men; (iii) HbA1c between 39–46 mmol/mol. Stage 2 (Metabolic Risk Factors and CKD): Encompasses individuals with metabolic diseases such as hypertriglyceridemia (≥1.7 mmol/L), hypertension, diabetes, metabolic syndrome, or chronic kidney disease classified as intermediate-to-high risk per KDIGO criteria. CKM stage 3 (high-risk subclinical disease): defined as very high-risk KDIGO CKD or an estimated 10-year CVD risk of ≥20% (8). This risk was assessed using the American Heart Association (AHA) PREVENT equations (15), chosen for their alignment with the CKM framework, integration of kidney function (eGFR), and coverage of the full spectrum of cardiovascular disease. Specific definitions for each risk factor and disease are provided in **Supplementary Table S1**.

### 2.5 Covariates

We adjusted for the following potential confounding factors: First, demographic factors, including age, gender (male, female), race (white, non-white), education level (college or university degree, non-college or non-university degree), income (Before tax ≤52,000 EUR, Before tax >52,000 EUR), and the Townsend Deprivation Index(an aggregate area-based measure of socioeconomic status). Second, health-related lifestyle variables: smoking status (never, former, current), alcohol consumption (yes, no), and sleep duration. Third, physical activity-related data: accelerometer wear time and accelerometer wear season (spring, summer, autumn, winter). Additionally, weekly MVPA duration was further adjusted in LPA analyzes, while weekly LPA duration was further adjusted in MVPA analyzes.

### 2.6 Statistical analyzes

Baseline characteristics are presented as mean ± standard deviation (SD) for continuous variables and as frequency (%) for categorical variables. The hazard ratio (HR) and 95% confidence interval (CI) for LPA and MVPA in relation to CVD incidence were estimated using Cox proportional hazards models. The proportional hazards assumption was assessed by inspecting Kaplan-Meier survival curves to ensure that the curves remained generally parallel and did not intersect over time. Three models were constructed: Model 1 was adjusted for demographic factors (age, sex, ethnicity, education level, and income); Model 2 additionally was adjusted for health-related lifestyle variables (smoking status, alcohol consumption, and sleep duration); Model 3 was further adjusted for accelerometer-based physical activity data (including accelerometer wear duration, wear season, and LPA or MVPA duration).

Restricted cubic splines (RCS) within the Cox proportional hazards model were used to evaluate the dose-response relationships of LPA and MVPA with the HR for CVD incidence in Model 3. A three-knot spline was applied, with knots placed at the 10th, 50th, and 90th percentiles. This knot selection was based on Harrell’s guidelines to provide sufficient mathematical flexibility to capture non-linear trends while avoiding model overfitting at the extreme tails of the physical activity distributions (16). To formally quantify the threshold effects, a two-piecewise linear regression model was constructed. After confirming the non-linearity of the association via a specific non-linear test (P < 0.05), a recursive grid-search algorithm was applied to identify the optimal inflection point. The distinct risk slopes (hazard changes per unit of physical activity) both before and after these inflection points were calculated. A log-likelihood ratio test (LRT) was then employed to confirm that the two-piecewise model provided a statistically superior fit compared to a standard linear model. Finally, 95% confidence intervals (CIs) were calculated to explicitly account for uncertainty in the estimated inflection points and their corresponding segmented slopes. Based on the prevalence of exposure and the HR for the outcome, we calculated the population attributable fraction (PAF), which was used to theoretically estimate the proportion of incident CVD cases that might be statistically attributable to insufficient physical activity. Specifically, it projects the theoretical reduction in CVD incidence if individuals in the lowest activity groups of LPA and MVPA had the same risk profile as those in the highest activity groups. Given the observational character of this study, these PAF values only represent theoretical projections of associated risk. Furthermore, for Model 3, a risk matrix analysis was conducted to examine the joint association of different intensity physical activities with the hazard ratio for CVD. The group with the lowest levels of both LPA and MVPA was set as the reference.

Additionally, we employed compositional data analysis (CoDA) to estimate the effect of reallocation time from LPA to MVPA on the HR for CVD across populations stratified by predicted risk (17). Accelerometer-measured time was categorized into four mutually exclusive components—sedentary behavior, sleep, LPA, and MVPA—forming a complete 24-hour movement composition. To overcome the limitations of traditional regression, these durations were expressed as proportions and transformed from a constrained simplex space into an unconstrained real space. Within this framework, we applied an isotemporal substitution model (ISM) (18). Using the compositional mean of the cohort’s 24-hour movement behaviors as the baseline, we simulated theoretical, model-based estimated associations for substituting LPA with MVPA. This reallocation was modeled in 1-minute increments up to a maximum of 400 minutes per week, a range deliberately chosen to encompass the full spectrum of observed activity differences and evaluate the dose-response trajectory even at extreme distributions.

In subgroup analyzes, participants were stratified by age (<60, ≥60 years), gender (male, female), educational level (college or university degree, non-college or non-university degree), income (Before tax ≤52,000 EUR, Before tax >52,000 EUR), Townsend deprivation index (>2.43, ≤2.43), smoking status (never, former, current), and alcohol consumption (yes, no). For each physical activity variable, the lowest quartile group served as the reference to calculate the HR with its 95% CI for the highest quartile group. The P value for interaction was calculated to examine whether the association between PA level and CVD incidence differed significantly across subgroups. To ensure the robustness of the findings, several sensitivity analyzes were performed. First, participants who developed CVD events within the first five years of follow-up were excluded to minimize potential reverse causation. Second, to address potential heterogeneity among cardiovascular outcomes, separate analyzes were conducted for major CVD subtypes, including ischemic heart disease (IHD), stroke, and heart failure (HF). Given the multiple comparisons involved in the subgroup and interaction tests, we did not apply formal multiplicity adjustments. Consequently, to acknowledge the increased risk of chance findings, the results of all subgroup and interaction analyzes are presented as exploratory and hypothesis-generating.

All statistical analyzes were conducted using R software (version 4.0.3). Specific R packages utilized for the analyzes included survival and survminer for Cox proportional hazards modeling, rms for restricted cubic splines, segmented for two-piecewise linear regression (inflection point analysis), and compositions for the compositional data analysis. A two-sided P-value < 0.05 was considered statistically significant for the primary analyzes.

## 3 Result

### 3.1 Baseline characteristics

This study included 88,281 participants without CVD at baseline, categorized into CKM stages 0–3: 17,345 in stage 0, 26,400 in stage 1, 41,942 in stage 2, and 2,594 in stage 3. The mean age at baseline was 56.07 (± 7.8) years, with 43.57% male and 91.08% White. During a median follow-up of 7.29 years, 7,514 participants developed CVD.

Baseline characteristics according to quartiles of LPA (<1637, 1637–<2069, 2069–<2551, and ≥2551 min/week) and MVPA (<112, 112–<231, 231–<401, and ≥401 min/week) are presented in **Table 1**. Participants with higher levels of either LPA or MVPA shared several characteristics: they were younger, included a higher proportion of never-smokers, and were more likely to be in CKM stage 0 or 1 and less likely to be in stage 2 or 3. Distinct characteristics were also observed: higher LPA was more common among females, those with lower education and income, non-current drinkers, and participants who wore the device more frequently in autumn. In contrast, higher MVPA was more common among males, those with higher education and income, current drinkers, and participants with higher device wear frequency in summer.

**Table 1:** Baseline characteristics of the study population.

Characteristic	Total	Physical activity (min/week)							
		LPA				MVPA			
		Q1 (<1637)	Q2 (1637-<2069)	Q3 (2069-<2551)	Q4 (≥2551)	Q1 (<112)	Q2 (112-<231)	Q3 (231-<401)	Q4 (≥401)
n	88281	22074	22074	22063	22070	22072	22077	22084	22048
Age (years, mean ± SD)	56.07 ± 7.8	55.96 ± 7.98	56.23 ± 7.78	56.4 ± 7.69	55.72 ± 7.73	57.11 ± 7.77	56.28 ± 7.83	55.65 ± 7.8	55.25 ± 7.69
Male, n(%)	38466 (43.57)	13641 (61.8)	10293 (46.63)	8096 (36.69)	6436 (29.16)	6880 (31.17)	8497 (38.49)	10087 (45.68)	13002 (58.97)
White, n(%)	80404 (91.08)	20155 (91.31)	20248 (91.73)	20151 (91.33)	19850 (89.94)	20230 (91.65)	20089 (91)	19980 (90.47)	20105 (91.19)
Education (College or university degree), n(%)	38259 (43.34)	10591 (47.98)	10087 (45.7)	9250 (41.93)	8331 (37.75)	7291 (33.03)	9106 (41.25)	10452 (47.33)	11410 (51.75)
Income (before tax >52,000 Euro), n(%)	27818 (31.51)	7816 (35.41)	7444 (33.72)	6659 (30.18)	5899 (26.73)	5293 (23.98)	6593 (29.86)	7655 (34.66)	8277 (37.54)
Townsend deprivation index (mean ± SD)^†^	-1.73 ± 2.81	-1.51 ± 2.96	-1.77 ± 2.8	-1.85 ± 2.74	-1.81 ± 2.73	-1.78 ± 2.78	-1.79 ± 2.78	-1.69 ± 2.83	-1.69 ± 2.86
Smoking									
Never, n(%)	50698 (57.43)	12486 (56.6)	12540 (56.8)	12662 (57.4)	13010 (59)	11949 (54.1)	12880 (58.3)	13080 (59.2)	12789 (58)
Previous, n(%)	31401 (35.57)	7755 (35.1)	8011 (36.3)	8000 (36.3)	7635 (34.6)	8023 (36.4)	7725 (35)	7641 (34.6)	8012 (36.3)
Current, n(%)	6150 (6.97)	1823 (8.3)	1517 (6.9)	1391 (6.3)	1419 (6.4)	2089 (9.5)	1467 (6.7)	1357 (6.2)	1237 (5.7)
Current alcohol drinker, n(%)	83387 (94.46)	20855 (94.48)	20981 (95.05)	20889 (94.68)	20662 (93.62)	20397 (92.41)	20836 (94.38)	21034 (95.25)	21120 (95.79)
Wear time(days, mean ± SD)	6.66 ± 0.65	6.63 ± 0.69	6.67 ± 0.63	6.68 ± 0.61	6.65 ± 0.68	6.63 ± 0.69	6.67 ± 0.64	6.67 ± 0.65	6.68 ± 0.64
Wear season									
Spring, n(%)	20048 (22.71)	4868 (22.05)	5081 (23.02)	5080 (23.02)	5019 (22.74)	4501 (20.39)	4753 (21.53)	5104 (23.11)	5690 (25.81)
Summer, n(%)	23243 (26.33)	5503 (24.9)	5643 (25.6)	5841 (26.5)	6256 (28.4)	5190 (23.5)	5585 (25.3)	5860 (26.5)	6608 (30.0)
Autumn, n(%)	26312 (29.8)	6712 (30.4)	6614 (30.0)	6581 (29.8)	6405(29.0)	6724 (30.5)	6706 (30.4)	6583 (29.8)	6299 (28.6)
Winter, n(%)	18678 (21.16)	4991 (22.6)	4736(21.5)	4561 (20.7)	4390 (19.9)	5657 (25.6)	5033 (22.8)	4537 (20.5)	3451 (15.6)
CKM syndrome Stage									
Stage 0, n(%)	17345(19.6)	3117 (14.12)	4100 (18.57)	4711 (21.35)	5417 (24.54)	2871 (13.01)	4060 (18.39)	4900 (22.19)	5514 (25.01)
Stage 1, n(%)	26400(29.9)	6187 (28.03)	6601 (29.90)	6810 (30.87)	6802 (30.82)	6402 (29.0)	6694 (30.32)	6594 (29.86)	6710 (30.43)
Stage 2, n(%)	41942(47.5)	11745 (53.21)	10690 (48.43)	10010 (45.37)	9497 (43.03)	11971 (54.24)	10710 (48.51)	9994 (45.25)	9267 (42.03)
Stage 3, n(%)	2594(2.9)	1025 (4.64)	683 (3.09)	532 (2.41)	354 (1.60)	828 (3.75)	613 (2.78)	596 (2.70)	557 (2.53)

Data are shown as mean ± SD for continuous variables and number (%) for categorical variables. Percentages might not add up to.

100% due to rounding. LPA, light-intensity physical activity; MVPA, moderate-to-vigorous-intensity physical activity; CKM, cardiovascular-kidney-metabolic;

†The Townsend Deprivation Index is an area-based measure of socioeconomic status. Higher (positive) scores indicate greater levels of material deprivation, whereas lower (negative) scores indicate relative affluence.

### 3.2 Association of LPA and MVPA with CVD incidence

#### 3.2.1 Quartile

**Figure 2:**
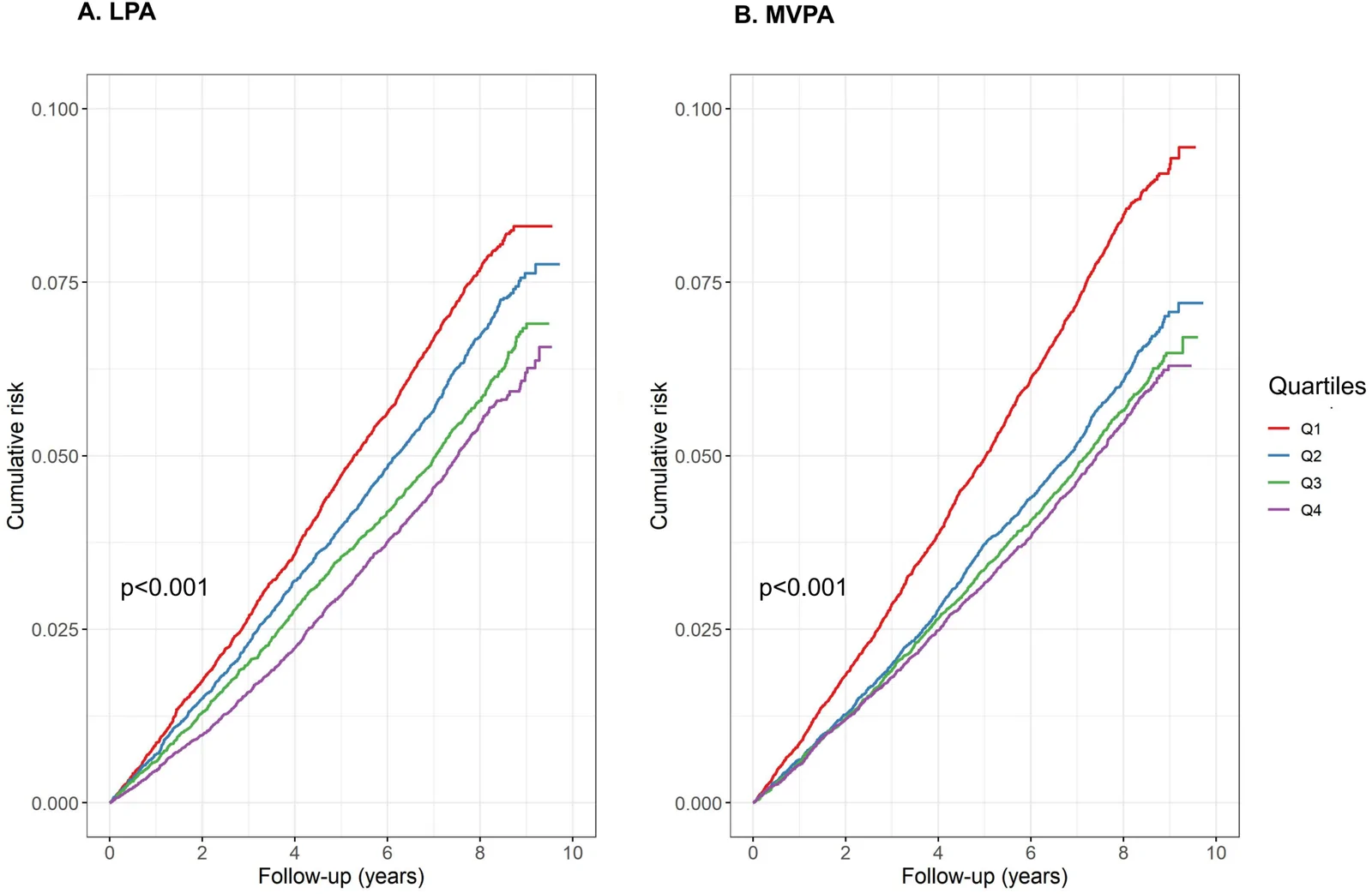
Cumulative risk of CVD incidence stratified by accelerometer-derived LPA and MVPA quartiles in CKM syndrome stage 0-3. **(A)** LPA, **(B)** MVPA. LPA, light-intensity physical activity; MVPA, moderate-to-vigorous-intensity physical activity; CVD, cardiovascular disease; CKM, cardiovascular-kidney-metabolic.

As shown in **Table 2**, among participants with CKM stages 0–3, compared to those in the lowest quartile of LPA (<1637 min/week), the HRs with 95% CIs for CVD were 0.96 (95% CI: 0.90–1.02), 0.87 (95% CI: 0.81–0.92), and 0.84 (95% CI: 0.79–0.91) for the groups with weekly LPA of 1,637–<2,069 min, 2,069–<2,551 min, and ≥2551 min, respectively (Model 3). Compared to participants in the lowest quartile of MVPA (<112 min/week), the HRs (95% CI) for CVD were 0.75 (95% CI: 0.71–0.80), 0.67 (95% CI: 0.63–0.72), and 0.60 (95% CI: 0.56–0.64) for the groups with weekly MVPA of 112–<231 min, 231–<401 min, and ≥401 min, respectively (Model 3). Furthermore, we observed that higher quartiles of both LPA and MVPA were associated with a reduction in the cumulative risk of CVD incidence (**Figure 2**).

**Table 2:** Associations of accelerometer-derived LPA and MVPA with CVD incidence in CKM syndrome stage 0-3.

	Physical activity category	No. of cases/total participants	Incidence rate per 1000 person-year	Hazard ratio (95% CI)		
				Model 1	Model 2	Model 3
LPA						
Q1	<1637min/week	2345/22074	8.16	Reference	Reference	Reference
Q2	1637-<2069min/week	1973/22074	6.80	0.91 (0.86-0.96)	0.91 (0.86-0.97)	0.96 (0.90-1.02)
Q3	2069-<2551min/week	1686/22063	5.78	0.82 (0.76-0.87)	0.82 (0.77-0.88)	0.87 (0.81-0.92)
Q4	≥2551min/week	1510/22070	5.15	0.80 (0.75-0.85)	0.81 (0.75-0.86)	0.84 (0.79-0.91)
MVPA						
Q1	<112min/week	2480/22072	8.64	Reference	Reference	Reference
Q2	112-<231min/week	1822/22077	6.27	0.73 (0.68-0.77)	0.74 (0.70-0.79)	0.75 (0.71-0.80)
Q3	231-<401min/week	1644/22084	5.63	0.65 (0.61-0.69)	0.66 (0.62-0.71)	0.67 (0.63-0.72)
Q4	≥401min/week	1568/22048	5.36	0.58 (0.54-0.62)	0.60 (0.56-0.64)	0.60 (0.56-0.64)

Model 1 was adjusted forage, sex, ethnic, qualification, income and Townsend deprivation index; Model 2 was adjusted for age, sex, ethnic, qualification, income, Townsend deprivation index, smoking, drinking and sleep time; Model 3 was adjusted for age, sex, ethnic, qualification, income, Townsend deprivation index, smoking, drinking, sleep time, wear time and season. Model 3 with LPA terms was additionally adjusted for total MVPA minutes per week, whereas the Model 3 with MVPA terms was additionally adjusted for total LPA minutes per week. 95% CI, 95% conﬁdence interval; CKM, cardiovascular-kidney-metabolic. LPA, light-intensity physical activity; MVPA, moderate-to-vigorous-intensity physical activity. LPA, light-intensity physical activity; MVPA, moderate-to-vigorous-intensity physical activity; CVD, cardiovascular disease; CKM, cardiovascular-kidney-metabolic.

When stratified by CKM stage, LPA showed the most pronounced risk reduction for CVD in individuals with stage 2 (**Supplementary Figure S1**; **Supplementary Tables S2-S5**). Compared to those with LPA <1580 min/week, participants with LPA ≥2493 min/week had a 18% lower risk (HR = 0.82; 95% CI: 0.75-0.90) (**Supplementary Table S4**). For MVPA, the most significant risk reductions were observed in individuals with CKM stage 1 and stage 2 (**Supplementary Figure S1**; **Supplementary Tables S2-S5**). In stage 1, compared to those with MVPA <115 min/week, participants with MVPA ≥405 min/week had a 42% lower CVD risk (HR = 0.58; 95% CI: 0.50–0.66) (**Supplementary Table S3**). In stage 2, compared to those with MVPA <98 min/week, participants with MVPA ≥374 min/week had a 33% lower risk (HR = 0.67; 95% CI: 0.61–0.73) (**Supplementary Table S4**).

#### 3.2.2 Volume

**Figure 3:**
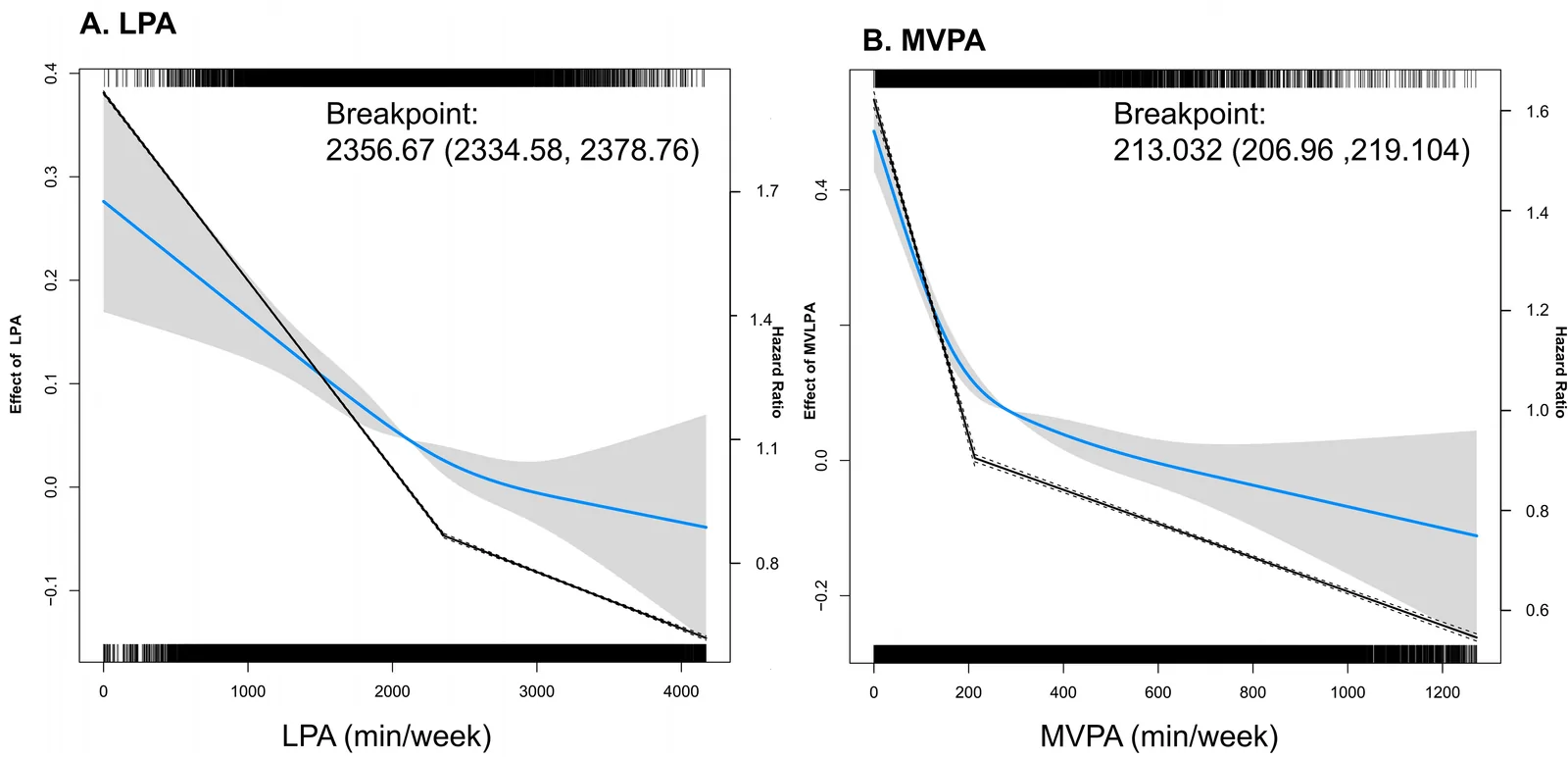
Inflection point analysis of accelerometer-derived LPA and MVPA with CVD incidence in CKM syndrome stage 0-3. **(A)** LPA, **(B)** MVPA. LPA, light-intensity physical activity; MVPA, moderate-to-vigorous-intensity physical activity; CVD, cardiovascular disease; CKM, cardiovascular-kidney-metabolic. The analysis is based on Model 3, which was adjusted for age, sex, ethnicity, qualification, income, Townsend deprivation index, smoking, drinking, sleep time, wear time, and season. Model 3 with LPA terms was additionally adjusted for total MVPA minutes per week, whereas Model 3 with MVPA terms was additionally adjusted for total LPA minutes per week.

In the overall analysis of participants across CKM stages 0–3, we observed nonlinear L-shaped dose-response relationships between both LPA and MVPA duration and the risk of CVD incidence across CKM stages 0–3 (P for nonlinearity < 0.05; **Supplementary Figures S2**, **S3**). The risk of CVD decreased with increasing LPA and MVPA duration, but the magnitude of reduction was limited. Specifically, an inflection point was identified at 2,357 (95% CI: 2335 - 2379) min/week for LPA and at 213 (95% CI: 207 - 219) min/week for MVPA in stages 0–3 general population (**Figure 3**). Beyond these inflection points, although the risk of CVD continued to decline with further increases in activity time, the rate of decrease slowed. The change in slope was more pronounced for MVPA, with the post-inflection slope being approximately one-tenth of the pre-inflection slope (**Supplementary Table S6**). In contrast to the overall L-shaped pattern, the stage-specific analysis revealed a distinct U-shaped dose-response pattern between MVPA and CVD risk uniquely among individuals in CKM stage 3 (P for nonlinearity = 0.022; **Supplementary Figure S2**). The risk of CVD began to increase when MVPA exceeded a threshold of 460 (95%CI: 449-472) min/week.

#### 3.2.3 Percent contribution

**Figure 4:**
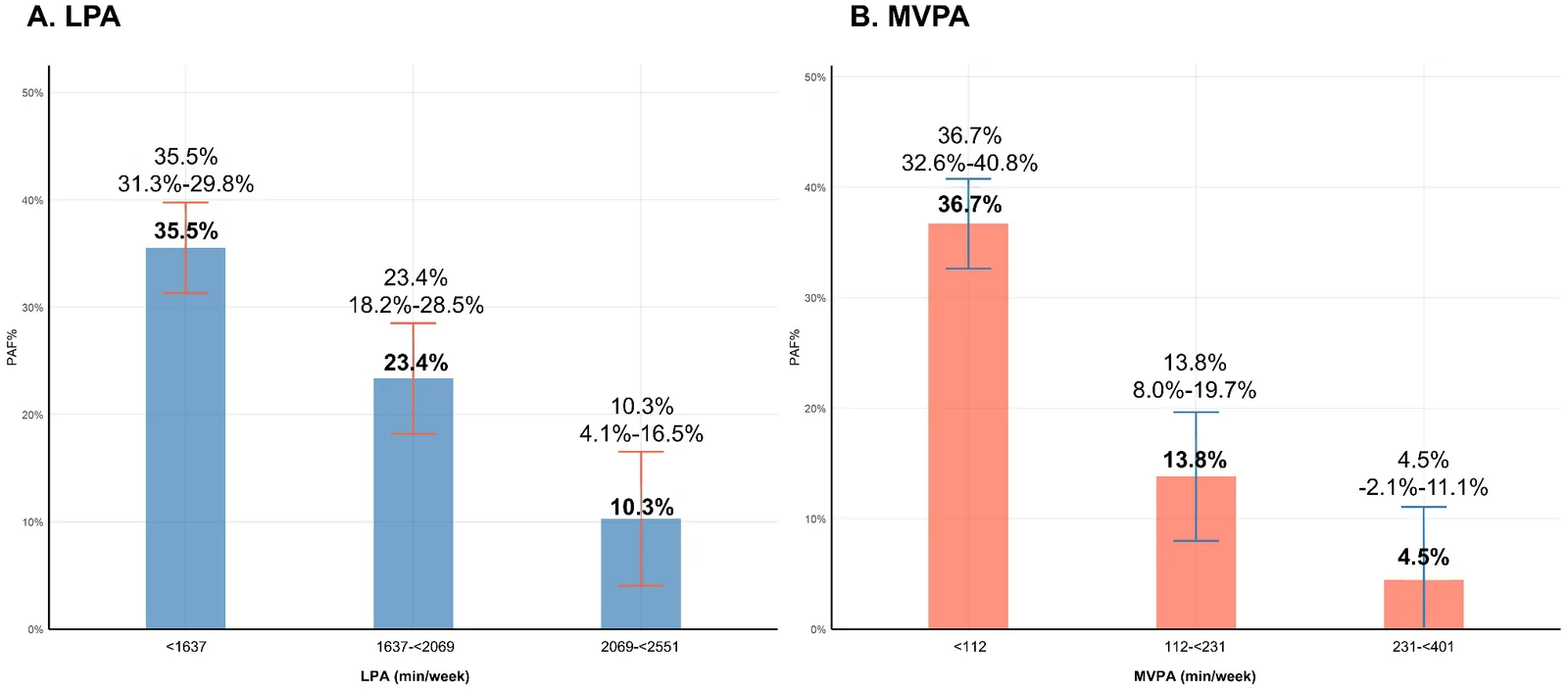
Population-attributable fractions of accelerometer-derived LPA and MVPA with CVD incidence in CKM syndrome stage 0-3. **(A)** LPA, **(B)** MVPA. LPA, light-intensity physical activity; MVPA, moderate-to-vigorous-intensity physical activity; CVD, cardiovascular disease; CKM, cardiovascular-kidney-metabolic. Error bars represent the 95% confidence intervals. The analysis is based on Model 3, which was adjusted for age, sex, ethnicity, qualification, income, Townsend deprivation index, smoking, drinking, sleep time, wear time, and season. Model 3 with LPA terms was additionally adjusted for total MVPA minutes per week, whereas Model 3 with MVPA terms was additionally adjusted for total LPA minutes per week.

A greater reduction in the attributable proportion of CVD incidence was observed when shorter durations of LPA and MVPA were replaced with longer durations (**Figure 4**, **Supplementary Figure S4**). Framed as modeled estimates under the assumption of causality, the PAF for CVD incidence decreased with increasing durations of both LPA and MVPA. Among participants with CKM stages 0–3, the PAF values were 35.5%, 23.4%, and 10.3% for those with weekly LPA durations of <1637 min, 1637–<2069 min, and 2069–<2551 min, respectively. For weekly MVPA durations of <112 min, 112–<231 min, and 231–<401 min, the corresponding PAF values were 36.7%, 13.8%, and 4.5%.

### 3.3 Joint association between LPA, MVPA with CVD incidence

**Figure 5:**
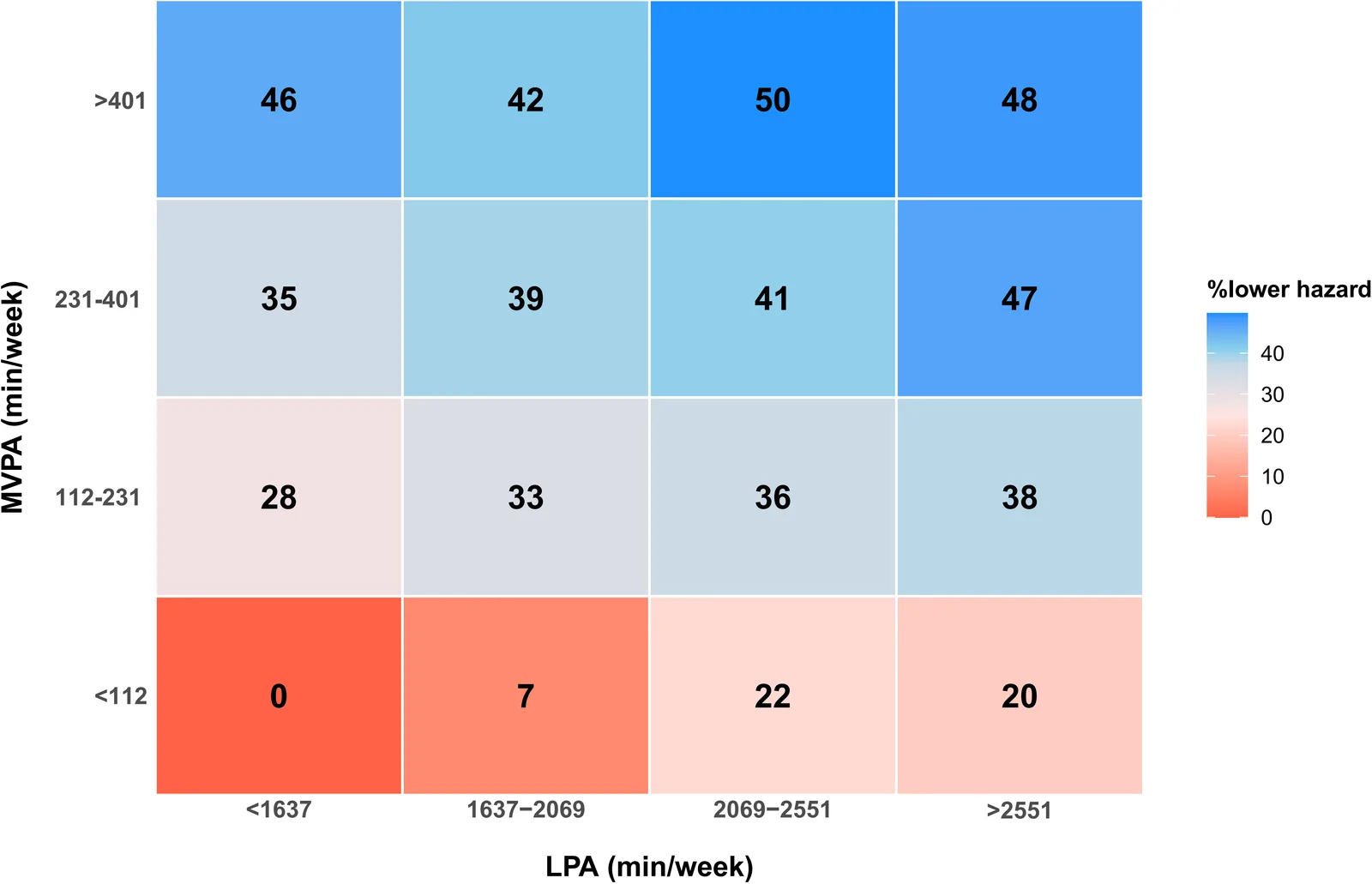
The risk matrix for the joint association of accelerometer-derived LPA and MVPA with CVD incidence in CKM syndrome stage 0-3. LPA, light-intensity physical activity; MVPA, moderate-to-vigorous-intensity physical activity; CVD, cardiovascular disease; CKM, cardiovascular-kidney-metabolic. Numbers in the matrix represent the percentage of risk reduction (%) compared with the reference group (the matrix marked ‘0’, representing the lowest quartiles of both LPA and MVPA). The analysis is based on Model 3, which was adjusted for age, sex, ethnicity, qualification, income, Townsend deprivation index, smoking, drinking, sleep time, wear time, and season. Model 3 with LPA terms was additionally adjusted for total MVPA minutes per week, whereas Model 3 with MVPA terms was additionally adjusted for total LPA minutes per week.

Nearly all combinations of LPA and MVPA were associated with a reduced risk of CVD incidence (**Figure 5**; **Supplementary Tables S7****–S11**). Descriptively, certain combinations of physical activity were associated with similarly low estimated risks for CVD: when LPA exceeded 2,551 min/week, maintaining MVPA at only 231–401 min/week (HR = 0.53 [95% CI: 0.47–0.60]) was associated with a risk reduction similar in magnitude to that of MVPA >401 min/week (HR = 0.52 [95% CI: 0.45–0.59]). Notably, for individuals with CKM stage 3, MVPA needed to be maintained within the third quartile to produce a substantial reduction in CVD risk (**Supplementary Figure S5**).

### 3.4 Relocation of LPA and MVPA with CVD incidence

**Figure 6:**
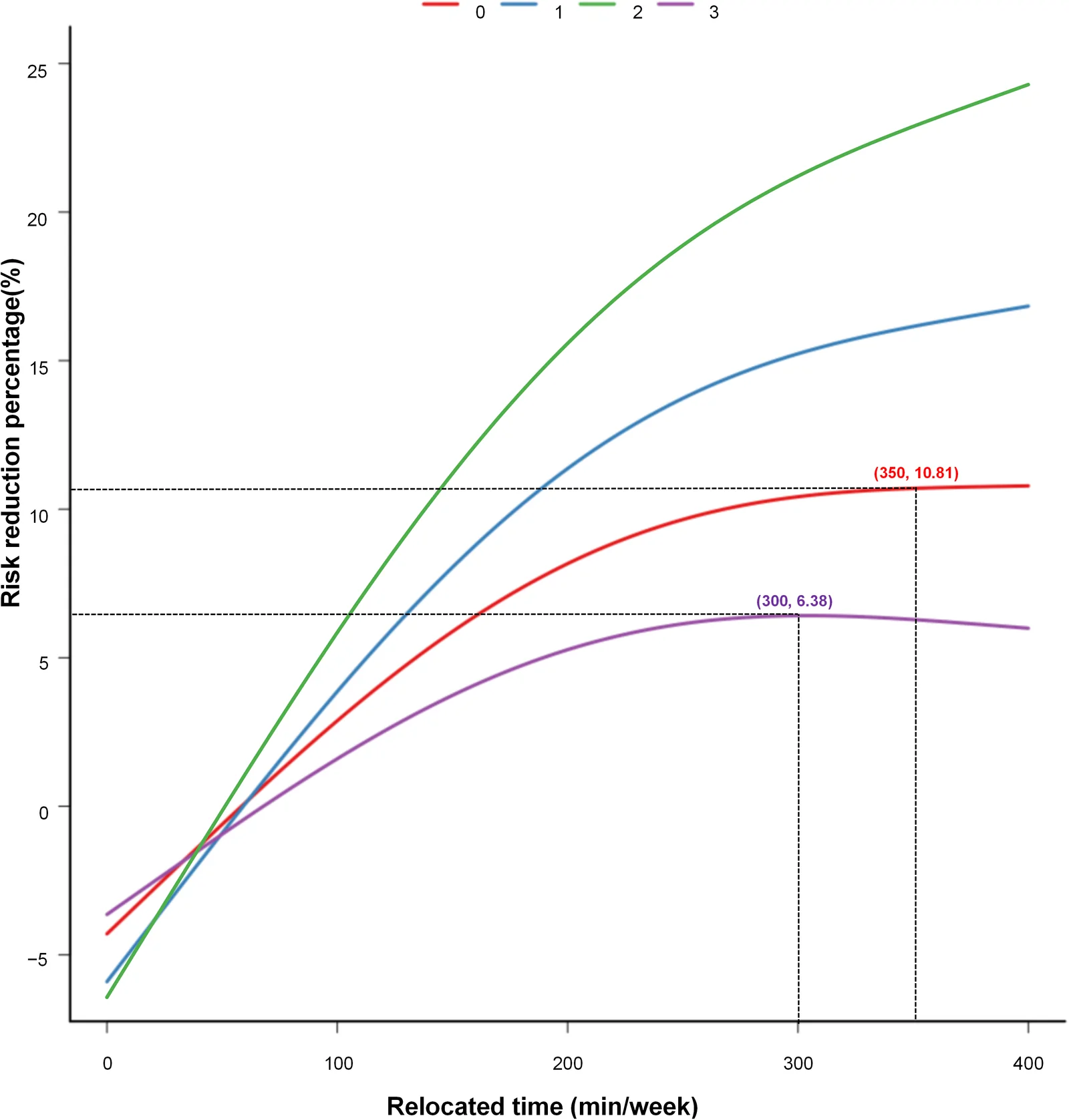
The CVD risk changes with reallocation of accelerometer-derived LPA and MVPA time to moderate-to-vigorous-intensity physical activity in CKM syndrome stage 0, 1, 2 and 3. LPA, light-intensity physical activity; MVPA, moderate-to-vigorous-intensity physical activity; CVD, cardiovascular disease; CKM, cardiovascular-kidney-metabolic. The analysis is based on Model 3, which was adjusted for age, sex, ethnicity, qualification, income, Townsend deprivation index, smoking, drinking, sleep time, wear time, and season. Model 3 with LPA terms was additionally adjusted for total MVPA minutes per week, whereas Model 3 with MVPA terms was additionally adjusted for total LPA minutes per week.

A greater theoretical reduction in estimated CVD incidence risk was associated with longer modeled durations of MVPA substituted for LPA (**Figure 6**). The estimated magnitude of risk reduction associated with the reallocation of time from LPA to MVPA in terms of reduced CVD incidence risk varied significantly across individuals with different CKM syndrome stages. The most pronounced modeled associations were observed for individuals in stages 2 and 1, where a longer duration of MVPA substituting for LPA was associated with a progressively greater reduction in estimated CVD risk. For stage 0 individuals, the modeled substitution of LPA with MVPA was associated with an estimated lower CVD risk of up to 10.81%; however, no further significant reduction estimated risk curve was observed once the weekly substitution exceeded 350 minutes. For stage 3 individuals, this theoretical substitution model indicated a maximum associated risk reduction of 6.38%, but the magnitude of this associated risk reduction began to decrease when the substitution duration exceeded 300 minutes per week.

### 3.5 Subgroup and sensitivity analyzes

In exploratory subgroup analyzes, the associations between LPA and MVPA against CVD incidence appeared to vary across specific populations (**Table 3**; **Supplementary Tables S12–S15**). Among individuals with CKM stages 0–3, higher levels of LPA were associated with a potentially greater reduction in estimated CVD risk in those aged >60 years compared with those ≤60 years (P for interaction = 0.001). Similarly, the inverse association for LPA appeared stronger in participants without a college or university degree than in those with the degree (P for interaction < 0.05). Regarding smoking status, higher LPA was linked to a more substantial decrease in CVD risk among current smokers than among former or never smokers (P for interaction = 0.01). For MVPA, stronger inverse association with CVD incidence was observed in females compared with males (P for interaction < 0.001), in non-drinkers compared with drinkers (P for interaction = 0.007), and in former smokers compared with current or never smokers (P for interaction < 0.05).

**Table 3:** Subgroup analysis of accelerometer-derived LPA and MVPA with CVD incidence in CKM syndrome stage 0-3 (highest versus lowest quarter of physical activity).

	LPA				MVPA			
Subgroup	Total participants	No. of cases	Hazard ratio (95% CI)	P for interaction	Total participants	No. of cases	Hazard ratio (95% CI)	P for interaction
Age								
>60 years	7637	854	0.778 (0.710-0.854)	0.001	7637	916	0.576 (0.528-0.630)	0.307
≤60 years	14429	683	0.863 (0.777-0.957)		14426	716	0.581 (0.526-0.642)	
Sex								
female	12453	640	0.750 (0.673-0.835)	0.377	12448	444	0.524 (0.468-0.587)	<0.001
male	9616	1045	0.861 (0.790-0.938)		9610	891	0.658 (0.605-0.716)	
Qualification								
Not College or university degree	12506	893	0.791 (0.725-0.865)	0.038	12505	1029	0.622 (0.573-0.676)	0.563
College or university degree	9565	591	0.881 (0.787-0.986)		9563	567	0.597 (0.535-0.666)	
Income								
Before tax ≤52,000 EUR	15115	1109	0.798 (0.737-0.865)	0.241	15113	1194	0.592 (0.549-0.639)	0.066
Before tax >52,000 EUR	6954	352	0.892 (0.772-1.031)		6952	397	0.669 (0.583-0.767)	
Townsend deprivation index								
>2.43	10938	764	0.846 (0.768-0.933)	0.064	10938	736	0.586 (0.532-0.644)	0.068
≤2.43	11121	743	0.800 (0.725-0.883)		11121	834	0.631 (0.575-0.692)	
Smoking								
Current	1537	121	0.657 (0.521-0.828)	0.01	1538	138	0.623 (0.499-0.779)	0.043
Never	12620	745	0.847 (0.766-0.937)		12624	793	0.642 (0.583-0.706)	
Previous	7850	651	0.860 (0.774-0.956)		7849	645	0.583 (0.526-0.646)	
Drinking								
No	1224	102	0.812 (0.623-1.058)	0.404	1222	87	0.403 (0.310-0.524)	0.007
Yes	20844	1402	0.828 (0.771-0.890)		20822	1474	0.619 (0.578-0.663)	

The analysis is based on Model 3, which was adjusted for age, sex, ethnicity, qualification, income, Townsend deprivation index, smoking, drinking, sleep time, wear time, and season. Model 3 with LPA terms was additionally adjusted for total MVPA minutes per week, whereas Model 3 with MVPA terms was additionally adjusted for total LPA minutes per week. LPA, light-intensity physical activity; MVPA, moderate-to-vigorous-intensity physical activity; CVD, cardiovascular disease; CKM, cardiovascular-kidney-metabolic.

In sensitivity analyzes, the inverse associations of LPA and MVPA with CVD incidence remained robust after excluding events occurring within the first five years of follow-up (**Supplementary Table S16**). When analyzing specific CVD subtypes, higher levels of both LPA and MVPA were generally associated with a reduced risk across all CVD-related outcomes. Specifically, higher MVPA levels showed significant protective associations with IHD (**Supplementary Table S17**), stroke (**Supplementary Table S18**), and HF (**Supplementary Table S19**) across all models. For LPA, significant inverse associations were observed for IHD and HF in the fully adjusted model (Model 3), while the association with stroke was significant in model 1 and 2 but became non-significant after further adjustment in Model 3.

## 4 Discussion

To our knowledge, this prospective study uniquely contributes to the recent cardiovascular-kidney-metabolic (CKM) literature by combining accelerometer-derived activity data with CKM stage 0–3 stratification within a large cohort. Our findings reveal that the duration and combination of LPA and MVPA exhibit distinct associations with incident CVD risk across these stages. Overall, LPA demonstrated the most pronounced inverse association with CVD in stage 2 individuals, while MVPA showed strong inverse associations in both stages 1 and 2, with estimated risk reductions increasing alongside activity duration. Compared with LPA, MVPA was associated with a greater magnitude of estimated risk reduction. Furthermore, modeling the theoretical substitution of LPA with MVPA was associated with progressively greater estimated benefits in stage 1 and 2 populations. However, for individuals already in stage 3, the inverse association of MVPA exhibited a threshold; exceeding a certain level was associated with an increased CVD risk. Similarly, the modeled risk reduction from the theoretical substitution of LPA with MVPA diminished beyond a specific duration in this population. These findings raise the possibility that the association between higher MVPA and CVD risk may differ in stage 3 and warrant further confirmation in future clinical trials. In stage 0 individuals, the associated cardiovascular risk reduction from theoretically substituting LPA with MVPA also plateaued beyond a certain threshold. Furthermore, we found that when total LPA was sufficient, maintaining MVPA at only the third quartile level was associated with estimated cardiovascular risks comparable to those of the highest quartile. These observational findings inform future hypothesis generation and contribute to the refinement of stage-specific observational evidence. By integrating device-measured activity with the CKM syndrome framework, our study complements recent translational lifestyle-medicine models and underscores the potential need for targeted, stage-specific exercise strategies.

Our study aligns with emerging literature that conceptualizes exercise as a core component of a broader lifestyle-medicine and translational-care framework across the CKM syndrome. For example, recent evidence from a longitudinal cohort study adopting the multi-state model analysis indicated that the highest level of physical activity could not only significantly reduce the risk of progression to the high-risk CKM stage (HR = 0.598; 95% CI: 0.459–0.777), but also substantially facilitate the reversal from the high-risk stage to the low-risk stage (HR = 2.995; 95% CI: 1.257–7.134) (19). Furthermore, emerging evidence derived from the All of Us cohort encompassing 17,000 adults enrolled by the National Institutes of Health demonstrates that achieving the highest level of physical activity is strongly associated with a substantial reduction in the risk of severe CKM syndrome (OR = 0.40; 95% CI: 0.31–0.53) (20). Moreover, exercise benefits vary by dose and pattern, with a nonlinear L-shaped association indicating moderate MVPA (600–12600 MET·min/week) forms the optimal protective range against early CKM risk (21). These emerging longitudinal and accelerometer-based findings align with our core observations. Furthermore, our data suggest that the associated cardiovascular benefits of physical activity may vary significantly depending on the disease stage.

Although stages 1–3 of CKM syndrome share common features of metabolic abnormalities, our study found a threshold effect for MVPA’s protective effect against CVD in stage 3 individuals, with risk increasing beyond a certain level. A recent study similarly suggested that higher intensities might not be suitable for adults with advanced (stage 3 or 4) CKM syndrome (22). Another study found that the threshold for achieving the optimal protective effect from MVPA was lower in stage 3 individuals compared to stages 0, 1, and 2, while the differences across stages for LPA were less pronounced. Compared to stages 0-2, stage 3 individuals have a higher 10-year CVD risk (23). A stratified study based on different CVD risk levels showed that the cardiovascular protective effect gained from equivalent MVPA gradually decreased from the low-to-moderate risk group to the very high-risk group (24). However, this observed threshold in stage 3 may reflect a complex mixture of biological heterogeneity within advanced disease states, residual confounding, and potential reverse causation. For example, undiagnosed subclinical conditions influence both high-intensity exercise behaviors and CVD outcomes. Furthermore, given the relatively smaller sample size in the stage 3 group, this finding is highly susceptible to instability at the extreme tails of the physical activity distribution. Therefore, the exact activity thresholds remain uncertain and should be viewed primarily as a hypothesis for future clinical investigations to explore. Our study also found that LPA was associated with a greater estimated reduction in CVD risk for stage 1 individuals, primarily characterized by excess adiposity and prediabetes, compared to stage 2. A four-arm randomized controlled crossover trial demonstrated that LPA in overweight or obese individuals significantly reduced blood glucose levels and improved heart rate variability (25). A cross-sectional study indicated that replacing sedentary time with LPA primarily contributed to a reduction in waist circumference, while replacing sedentary time with MVPA led to decreases in both BMI and blood lipids (26). These distinct associations highlight why Stage 1 and Stage 2 populations require individualized behavioral strategies. In Stage 1, the primary pathophysiological target is managing adiposity and increasing daily basal energy expenditure. For these individuals, accumulating a high volume of LPA is highly effective for fat oxidation and offers a sustainable, low-barrier behavioral starting point that maximizes long-term exercise adherence while minimizing injury risk (4, 27). However, as individuals progress to Stage 2, the clinical profile shifts toward established metabolic diseases, such as entrenched insulin resistance, dyslipidemia, and endothelial dysfunction. Reversing these more advanced impairments requires a stronger physiological stimulus. Incorporating MVPA becomes necessary in Stage 2 because higher-intensity exertion is physiologically required to effectively promote GLUT4 translocation for insulin-independent glucose uptake (28). Consequently, our findings support the individualization of CKM lifestyle intervention: for stage 1 individuals, maintaining a sufficient duration of LPA alone can provide significant cardiovascular protection, whereas for those progressing to stage 2, incorporating a certain duration of MVPA is necessary.

To further assess the impact of other factors on the association between LPA, MVPA, and CVD incidence, we conducted subgroup analyzes. The study found that the beneficial effects of LPA on CVD incidence were more significant in individuals aged >60 years and current smokers, while MVPA was more significant in females and previous smokers. A cohort study based on UK Biobank similarly showed that LPA had a significant risk-reducing effect in individuals >60 years old, with no significant protective association observed in those ≤60 years old (29). A study based on the Swedish SNAC-K study found that in the older age group, each additional 100 LPA walking activities reduced CVD risk by 39% (0.61; 95% CI 0.39–0.95), whereas no significant association was found in younger older adults. This may be because muscle and bone mass in older adults peak in early adulthood and then decline with age, leading to decreased muscle strength and physical function, which in turn results in longer sedentary time among the elderly (30). As suggested by WHO guidelines, replacing sedentary time with physical activities, including LPA, in the elderly population can yield substantial health benefits (1). Beyond physiological factors, psychosocial determinants of exercise adherence help explain the pronounced cardiovascular benefits of LPA in older adults. Specifically, compared to higher-intensity exercises, LPA requires significantly lower levels of mental toughness and behavioral persistence, making it highly accessible and easier for the elderly to execute daily (31). Additionally, we observed gender differences in the benefits of MVPA. Existing evidence indicates that although males typically possess greater absolute exercise capacity, females have inherent physiological advantages, including higher skeletal muscle capillary density and a greater proportion of type I muscle fibers (32). These characteristics enhance blood perfusion and metabolic efficiency during exercise, potentially promoting better vascular function responses to PA in females. Notably, LPA and MVPA were associated with lower CVD incident risk in current and previous smokers. Smoking initiates the atherosclerotic process by impairing endothelial function: harmful substances in cigarette smoke (e.g., carbon monoxide, nicotine) reduce the bioavailability of vascular endothelial nitric oxide, increase the expression of adhesion molecules, and promote the adhesion of platelets and macrophages, thereby creating a pro-coagulant and pro-inflammatory environment (33, 34). In contrast, physical activity promotes the complete mechanism of vascular endothelial repair by upregulating the expression of nitric oxide synthase in the vascular endothelium (35). A 5-year follow-up study showed that higher baseline daily physical activity levels were significantly associated with better endothelial function (assessed by reactive hyperemia index) after 5 years (36). A similar study indicates that current or former smokers and excessive drinkers could gain more benefits from higher physical activity intensity to reduce the risk of cardiometabolic multimorbidity progression and death (37).

The association between physical activity and CVD risk in individuals with CKM syndrome stages 0–3 may be explained by multiple potential mechanisms. Stage 0 individuals have no CKM risk factors, and the prevention goal is to achieve and maintain ideal health by adhering to the “Essential 8,” with physical activity being one of the eight core components (38). Stage 1 is characterized by overweight/obesity or impaired glucose tolerance. Exercise can reverse the inflammatory state of white adipose tissue, reduce infiltration of M1-type macrophages and secretion of pro-inflammatory factors (TNF-α, IL-6), thereby improving insulin resistance (39). Stage 2 patients already have hypertension, diabetes, or early-stage CKD. Physical activity at this stage was associated with multi-target organ protective effects: moderate-intensity aerobic exercise increases renal blood flow, reduces intraglomerular pressure, and delays the progression of proteinuria (40, 41); exercise upregulates endothelial nitric oxide synthase (eNOS) expression, improves vascular dilation function, and inhibits vascular inflammation and oxidative stress. Studies show that after 4 weeks of exercise training, eNOS protein expression in endothelial cells of the left internal mammary artery (LIMA) was upregulated 2-fold, and phosphorylation levels at the key activation site Ser increased 4-fold (42). Clear evidence supports that exercise can directly downregulate blood lipid levels: a meta-analysis incorporating 148 RCTs showed that each additional session of aerobic exercise per week reduced total cholesterol by 7.68 mg/dL, lowered TC by 0.5 mg/dL, and increased HDL by 2.11 mg/dL. For stage 3 individuals, physical activity can reverse early vascular and myocardial pathology: exercise reduces matrix metalloproteinase (MMP) activity and increases fibrous cap thickness (43). An experimental study showed that exercise training increased fibrous cap thickness in plaques of diabetic apoE-/- mice and significantly reduced the positive staining area for proteases such as MMP-2, MMP-3, and MMP-8 within the plaques. Exercise can also reduce myocardial stiffness and improve left ventricular diastolic function (44). Regular exercise can also decrease levels of markers such as BNP and NT-proBNP (45, 46).

Our study comprehensively investigates the association between LPA, MVPA and the CVD incidence risk in CKM syndrome stages 0–3 individuals. The use of objective accelerometer-measured data significantly reduces recall bias and social desirability bias compared to traditional self-reports. By excluding participants with baseline CVD, censoring those who developed outcomes within 2 years, and extensively adjusting for potential confounders, the risks of reverse causality were effectively mitigated, enhancing the reliability of the findings. The large sample size and consistent sensitivity analyzes further support the robustness of the study results. This study also employed multiple analytical approaches, including inflection point analysis, joint analysis, and isotemporal substitution models based on compositional data analysis, to comprehensively assess the impact of LPA and MVPA duration, combinations, and substitution on CVD incidence risk among individuals at different stages of CKM syndrome. Nonetheless, this study has several limitations. First, the representativeness and generalizability of the study population are limited: the research is based on the UK Biobank, where participants are predominantly middle-aged to older adults of White ethnicity. Therefore, caution is needed when generalizing the conclusions to other populations. Future validation in more diverse populations is warranted. Second, potential biases in measurement and classification: although accelerometers provide objective measurement, the study relied on a single time-point assessment of activity, failing to capture changes in physical activity over time. This nondifferential misclassification may lead to an underestimation of the associations. Third, composite CVD outcomes may obscure the heterogeneity among different disease types, which needs to be clarified by future studies focusing on specific CVD diseases. Fourth, it should be noted that while the official CKM framework encompasses stages 0–4, our analysis was restricted to stages 0–3 (as individuals with baseline CVD were excluded to assess incident risk). Fifth, the smaller sample size in specific subgroups, particularly in CKM stage 3, reduces statistical power; consequently, the nonlinear findings observed within this advanced stage may be less stable and should be interpreted carefully. Sixth, despite adjusting for a comprehensive array of established covariates, we cannot rule out the possibility of residual confounding. Variables such as nuanced dietary patterns, baseline functional fitness, healthcare utilization, and specific pharmacological treatments represent potential sources of unmeasured confounding that could impact the reported effect estimates. Additionally, relying solely on the visual inspection of Kaplan-Meier curves to assess the proportional hazards assumption, rather than employing formal statistical tests like Schoenfeld residuals, represents a limitation of our statistical validation approach. Given these methodological and population constraints, the translational clinical implications of our findings must remain cautious until further validated.

## 5 Conclusion

This population-based cohort study, utilizing device-measured data, suggests that the association between physical activity and CVD risk varies among individuals at different stages of CKM syndrome. For individuals in stages 1–2, higher levels of LPA and MVPA were associated with a lower risk of incident CVD. Conversely, for individuals in stage 3, we observed a potential non-linear trend where CVD risk appeared to increase after MVPA exceeded a certain threshold. However, this specific finding is subject to greater statistical uncertainty and warrants cautious interpretation. Overall, these findings highlight that the cardiovascular benefits of physical activity may be stage-dependent. While our results underscore the potential value of considering CKM stages when evaluating lifestyle factors, further robust research is required to clarify these relationships—particularly in advanced stages—before these observational findings can be translated into prescriptive clinical guidelines.

## Data Availability

This research was conducted using data from the UK Biobank resource under Application Number [ID: 906,426]. Due to the strict data sharing policies and ethical agreements of the UK Biobank, the authors are not permitted to share the individual-level data underlying this study. However, the data are available to all bona fide researchers upon approval of an application to the UK Biobank (https://www.ukbiobank.ac.uk/). Requests to access the datasets should be directed to J-QA, m13120067887@163.com.
