# Correction: Association of accelerometer-measured physical activity and the risk of incident cardiovascular diseases within the AHA cardiovascular-kidney-metabolic syndrome 0–4 framework: a prospective cohort of the UK Biobank

**DOI:** 10.3389/fendo.2026.2007019

**Published:** 2026-10-06

**Authors:** Jun-Qiao An, Xue He, Jing-Yi Ding, Yan Wu, Xiang-Wei Luan, Zhao-Zong Xu, Tian-Tian Guo, Qing-Yong He

**Affiliations:** 1 Beijing University of Chinese Medicine, Beijing, China; 2 Guang’anmen Hospital, China Academy of Chinese Medical Sciences, Beijing, China

**Keywords:** dose-response relationship, isotemporal substation models, lifestyle medicine, multimorbidity, wearable electronic devices

The funder “Young Beijing Scholar Program (No. 075); the National Natural Science Foundation of China (No. 82575044) and the Special Program for Cultivating Outstanding Young Scientific and Technological Talents of China Academy of Chinese Medical Sciences (No. CI2023D004)” to “Qing Yong He” was erroneously omitted.

The original version of this article has been updated.

