# Supplementary material for: Association of accelerometer-measured physical activity and the risk of incident cardiovascular diseases within the AHA cardiovascular-kidney-metabolic syndrome 0–4 framework: a prospective cohort of the UK Biobank
Source: Front Endocrinol (Lausanne). 2026 Jul 31;17:1844167. doi: 10.3389/fendo.2026.1844167 (PMC13472813; doi:10.3389/fendo.2026.1844167)
Supplement: Supplementary file 1 [file DataSheet1.docx]

**Supplementary Materials**

[Table S1. Definition of CKM syndrome.	2](#_Toc27553)

[Table S2. Associations of accelerometer-derived LPA and MVPA categories with CVD incidence in CKM syndrome Stage 0.	4](#_Toc24466)

[Table S3. Associations of accelerometer-derived LPA and MVPA categories with CVD incidence in CKM syndrome Stage 1.	5](#_Toc5060)

[Table S4. Associations of accelerometer-derived LPA and MVPA categories with CVD incidence in CKM syndrome Stage 2.	6](#_Toc17296)

[Table S5. Associations of accelerometer-derived LPA and MVPA categories with CVD incidence in CKM syndrome Stage 3.	7](#_Toc23606)

[Table S6. Inflection point analysis of accelerometer-derived LPA and MVPA with CVD incidence in CKM syndrome stage 0-3.	8](#_Toc12946)

[Table S7. The risk matrix for the joint association of accelerometer-derived LPA and MVPA with CVD incidence in CKM syndrome stage 0-3	9](#_Toc17733)

[Table S8. The risk matrix for the joint association of accelerometer-derived LPA and MVPA with CVD incidence in CKM syndrome stage 0	10](#_Toc10186)

[Table S9. The risk matrix for the joint association of accelerometer-derived LPA and MVPA with CVD incidence in CKM syndrome stage 1	11](#_Toc23697)

[Table S10. The risk matrix for the joint association of accelerometer-derived LPA and MVPA with CVD incidence in CKM syndrome stage 2.	12](#_Toc25763)

[Table S11. The risk matrix for the joint association of accelerometer-derived LPA and MVPA with CVD incidence in CKM syndrome stage 3.	13](#_Toc26343)

[Table S12. Subgroup analysis of accelerometer-derived LPA and MVPA with CVD incidence in CKM syndrome stage 0.	14](#_Toc10146)

[Table S13. Subgroup analysis of accelerometer-derived LPA and MVPA with CVD incidence in CKM syndrome stage 1.	15](#_Toc2576)

[Table S14. Subgroup analysis of accelerometer-derived LPA and MVPA with CVD incidence in CKM syndrome stage 2.	16](#_Toc17235)

[Table S15. Subgroup analysis of accelerometer-derived LPA and MVPA with CVD incidence in CKM syndrome stage 3.	17](#_Toc19089)

[Table S16. Sensitivity analysis of accelerometer-derived LPA and MVPA categories with CVD incidence in CKM syndrome Stage 0-3, excluding events occurring within the first five years of follow-up	18](#_Toc23984)

[Table S17. Sensitivity analysis of accelerometer-derived LPA and MVPA categories with IHD incidence in CKM syndrome Stages 0-3	19](#_Toc9209)

[Table S18. Sensitivity analysis of accelerometer-derived LPA and MVPA categories with stroke incidence in CKM syndrome Stages 0-3	20](#_Toc9119)

[Table S19. Sensitivity analysis of accelerometer-derived LPA and MVPA categories with HF incidence in CKM syndrome Stages 0-3	21](#_Toc30943)

[Figure S1. Cumulative risk of CVD incidence stratified by accelerometer-derived LPA and MVPA quartiles in CKM syndrome stage 0, 1, 2 and 3	22](#_Toc22903)

[Figure S2. Restricted Cubic Splines of accelerometer-derived LPA and MVPA with CVD incidence in CKM syndrome stage 0, 1, 2, 3 and 0-3.	23](#_Toc16342)

[Figure S3. Inflection point analysis of accelerometer-derived LPA and MVPA with CVD incidence in CKM syndrome stage 0, 1, 2, 3 and 0-3	24](#_Toc14518)

[Figure S4. Population-attributable fractions of accelerometer-derived LPA and MVPA with CVD incidence in CKM syndrome stage 0, 1, 2 and 3	25](#_Toc15568)

[Figure S5. The risk matrix for the joint association of accelerometer-derived LPA and MVPA with CVD incidence in CKM syndrome stage 0, 1, 2 and 3.	26](#_Toc21195)

## Table S1. Definition of CKM syndrome.

| **Stage** | **Definition** |
| --- | --- |
| **0** | Normal BMI  (Asian＜23 kg/m^2^, Other races and ethnicities＜25 kg/m^2^)  Normal waist circumference  (Waist circumference＜80/90 cm in Asian women/men, Waist circumference＜88/102cm in women and men of other races and ethnicities)  And participants who do not meet the standards of other stages |
| **1** | Elevated BMI  (Asian≥23 kg/m^2^, Other races and ethnicities≥25 kg/m^2^)  Elevated waist circumference  (Waist circumference ≥ 80/90cm in Asian women/men, Waist circumference ≥88/102cm in women and men of other races and ethnicities)  Or prediabetes  (HbA1c between 5.7% and 6.5% or Fasting blood glucose between 100mg/dL and 126 mg/dL) |
| **2** | Participants with metabolic risk factors or those with moderate-to-high risk CKD according to KDIGO criteria  Metabolic risk factors that meet the criteria include: Elevated fasting serum triglycerides(≥135 mg/dL), Hypertension, Diabetes or Metabolic Syndrome (The following≥3 items: Elevated waist circumference, Low HDL levels(<40/50 mg/dL in men and women), Fasting Serum Triglycerides≥150 mg/dL, Elevated blood pressure(SBP≥130, DBP≥80 mmHg, and/or using antihypertensive drugs  ) or prediabetes)  Moderate-to-high risk CKD  Determine CKD stage using eGFR and ACR, the calculation method of eGFR adopts New Creatinine- and Cystatin C-Based Equations to Estimate GFR without Race^[1]^.  Moderate risk: eGFR: 45-59ml/min/1.73m²; and  ACR: 30-299mg/g or 3-29mg/mmol  high risk: eGFR: 30-44ml/min/1.73m²; and  ACR≥30mg/g or≥3mg/mmol |

| **3** | CKM Stage 3 was identified based of the presence of very-high-risk KDGIO CKD stages1 or a high-predicted 10-year CVD risk. 10-year cardiovascular risk was estimated with the AHA Predicting Risk of CVD EVENTs (PREVENT) equations. High risk was defined as ≥20% 10-year CVD risk. The PREVENT equations were developed and validated for adults 30-79 years of age. Further, PREVENT was developed for variables with the following ranges: total cholesterol 130-320 mg/dL, HDL 20-100 mg/dL, systolic blood pressure 90-200 mmHg, and GFR 14-140 mL/min/1.73m2. To approximate PREVENT risk strata, values for these variables above or below these bounds were set to the upper or lower bounds of allowable values respectively (for example, total cholesterol of 330 mg/dL was set as 320 mg/dL). |
| --- | --- |
| **4** | Confirmed cardiovascular disease  (CHD, AP, Heart attack, HF, AF, Peripheral artery disease and stroke) |

Notes: CKM: cardiovascular-kidney-metabolic, BMI: body mass index, HbA1c: Glycated hemoglobin A1c, CKD: chronic kidney disease, HDL: High Density Lipoprotein, SBP: Systolic blood pressure, DBP: Diastolic blood pressure, eGFR: estimated glomerular filtration rate, ACR: urine protein-to-creatinine ratio, CHD: Coronary Heart Disease, AP: Angina pectoris, HF: Heart failure, AF: atrial fibrillation; KDIGO: Kidney Disease: Improving Global Outcomes; AHA: American Heart Association; PREVENT: Predicting Risk of Cardiovascular disease EVENTs

## Table S2. Associations of accelerometer-derived LPA and MVPA categories with CVD incidence in CKM syndrome Stage 0. 95% CI, 95% confidence interval; LPA, light-intensity physical activity; MVPA, moderate-to-vigorous-intensity physical activity; CVD, cardiovascular disease; CKM, cardiovascular-kidney-metabolic.

| Level | physical activity  category | No. of cases/total  participants | Incidence rate per 1000 person-year |  | Hazard ratio (95% CI) |  |
| --- | --- | --- | --- | --- | --- | --- |
|  |  |  |  | Model 1 | Model 2 | Model 3 |
| LPA |  |  |  |  |  |  |
| Q1 | <1780min/week | 215/4339 | 3.68 | Reference | Reference | Reference |
| Q2 | 1780-<2211min/week | 212/4334 | 3.63 | 1.01 (0.83-1.22) | 1.02 (0.84-1.24) | 1.03 (0.85-1.25) |
| Q3 | 2211-<2687min/week | 176/4338 | 3.01 | 0.85 (0.69-1.05) | 0.87 (0.70-1.07) | 0.88 (0.71-1.08) |
| Q4 | ≥2687min/week | 175/4334 | 2.99 | 0.91 (0.74-1.12) | 0.94 (0.76-1.17) | 0.94 (0.75-1.16) |
| MVPA |  |  |  |  |  |  |
| Q1 | <155min/week | 227/4337 | 3.89 | Reference | Reference | Reference |
| Q2 | 155-<284min/week | 194/4336 | 3.32 | 0.87 (0.71-1.05) | 0.89 (0.73-1.07) | 0.89 (0.74-1.08) |
| Q3 | 284-<458min/week | 194/4336 | 3.33 | 0.83 (0.68-1.01) | 0.85 (0.70-1.03) | 0.85 (0.70-1.04) |
| Q4 | ≥458min/week | 163/4336 | 2.78 | 0.63 (0.51-0.77) | 0.65 (0.53-0.80) | 0.65 (0.53-0.81) |

Note: The quartile cut points for LPA and MVPA in this table are calculated based on the activity distribution of participants strictly within CKM Stage 0.   Supplementary materials: Confirm all supplementary tables and figures carry complete standalone legends. Model 1 was adjusted for age, sex, ethnic, qualification, income and Townsend deprivation index; Model 2 was adjusted for age, sex, ethnic, qualification, income, Townsend deprivation index, smoking, drinking and sleep time; Model 3 was adjusted for age, sex, ethnic, qualification, income, Townsend deprivation index, smoking, drinking, sleep time, wear time and season. Model 3 with LPA terms was additionally adjusted for total MVPA minutes per week, whereas the Model 3 with MVPA terms was additionally adjusted for total LPA minutes per week.

## Table S3. Associations of accelerometer-derived LPA and MVPA categories with CVD incidence in CKM syndrome Stage 1. 95% CI, 95% conﬁdence interval; LPA, light-intensity physical activity; MVPA, moderate-to-vigorous-intensity physical activity; CVD, cardiovascular disease; CKM, cardiovascular-kidney-metabolic.

Note: The quartile cut points for LPA and MVPA in this table are calculated based on the activity distribution of participants strictly within CKM Stage 1. Model 1 was adjusted for age, sex, ethnic, qualification, income and Townsend deprivation index; Model 2 was adjusted for age, sex, ethnic, qualification, income, Townsend deprivation index, smoking, drinking and sleep time; Model 3 was adjusted for age, sex, ethnic, qualification, income, Townsend deprivation index, smoking, drinking, sleep time, wear time and season. Model 3 with LPA terms was additionally adjusted for total MVPA minutes per week, whereas the Model 3 with MVPA terms was additionally adjusted for total LPA minutes per week.

| Level | physical activity  category | No. of cases/total  participants | Incidence rate per 1000 person-year |  | Hazard ratio (95% CI) |  |
| --- | --- | --- | --- | --- | --- | --- |
|  |  |  |  | Model 1 | Model 2 | Model 3 |
| LPA |  |  |  |  |  |  |
| Q1 | <1667min/week | 512/6600 | 5.88 | Reference | Reference | Reference |
| Q2 | 1667-<2095min/week | 509/6601 | 5.84 | 1.04 (0.92-1.17) | 1.06 (0.94-1.20) | 1.10(0.97-1.25) |
| Q3 | 2095-<2568min/week | 393/6599 | 4.47 | 0.85 (0.74-0.97) | 0.87 (0.76-1.00) | 0.91(0.80-1.05) |
| Q4 | ≥2568min/week | 360/6600 | 4.08 | 0.85 (0.74-0.97) | 0.89 (0.77-1.03) | 0.92(0.80-1.07) |
| MVPA |  |  |  |  |  |  |
| Q1 | <115min/week | 568/6603 | 6.53 | Reference | Reference | Reference |
| Q2 | 115-<233min/week | 415/6597 | 4.73 | 0.69 (0.61-0.79) | 0.70 (0.62-0.80) | 0.71(0.62-0.80) |
| Q3 | 233-<405min/week | 405/6604 | 4.62 | 0.67 (0.59-0.76) | 0.68 (0.60-0.78) | 0.68(0.60-0.77) |
| Q4 | ≥405min/week | 386/6596 | 4.39 | 0.57 (0.49-0.65) | 0.58 (0.51-0.67) | 0.58(0.50-0.66) |

## Table S4. Associations of accelerometer-derived LPA and MVPA categories with CVD incidence in CKM syndrome Stage 2. 95% CI, 95% confidence interval; LPA, light-intensity physical activity; MVPA, moderate-to-vigorous-intensity physical activity; CVD, cardiovascular disease; CKM, cardiovascular-kidney-metabolic.

| Level | physical activity  category | No. of cases/total  participants | Incidence rate per 1000 person-year |  | Hazard ratio (95% CI) |  |
| --- | --- | --- | --- | --- | --- | --- |
|  |  |  |  | Model 1 | Model 2 | Model 3 |
| LPA |  |  |  |  |  |  |
| Q1 | <1580min/week | 1312/10493 | 9.69 | Reference | Reference | Reference |
| Q2 | 1580-<2008min/week | 1076/10480 | 7.86 | 0.85 (0.78-0.92) | 0.85 (0.78-0.92) | 0.88(0.82-0.96) |
| Q3 | 2008-<2493min/week | 1000/10483 | 7.30 | 0.81 (0.74-0.88) | 0.80 (0.74-0.87) | 0.84(0.77-0.92) |
| Q4 | ≥2493min/week | 923/10486 | 6.70 | 0.79 (0.72-0.86) | 0.78 (0.71-0.85) | 0.82(0.75-0.90) |
| MVPA |  |  |  |  |  |  |
| Q1 | <98min/week | 1346/10523 | 9.92 | Reference | Reference | Reference |
| Q2 | 98-<211min/week | 1050/10468 | 7.69 | 0.75 (0.69-0.82) | 0.76 (0.70-0.83) | 0.78(0.72-0.84) |
| Q3 | 211-<374min/week | 967/10480 | 7.05 | 0.69 (0.63-0.75) | 0.70 (0.64-0.76) | 0.71(0.65-0.78) |
| Q4 | ≥374min/week | 948/10471 | 6.88 | 0.64 (0.59-0.70) | 0.65 (0.60-0.71) | 0.67(0.61-0.73) |

Note: The quartile cut points for LPA and MVPA in this table are calculated based on the activity distribution of participants strictly within CKM Stage 2. Model 1 was adjusted for age, sex, ethnic, qualification, income and Townsend deprivation index; Model 2 was adjusted for age, sex, ethnic, qualification, income, Townsend deprivation index, smoking, drinking and sleep time; Model 3 was adjusted for age, sex, ethnic, qualification, income, Townsend deprivation index, smoking, drinking, sleep time, wear time and season. Model 3 with LPA terms was additionally adjusted for total MVPA minutes per week, whereas the Model 3 with MVPA terms was additionally adjusted for total LPA minutes per week.

## Table S5. Associations of accelerometer-derived LPA and MVPA categories with CVD incidence in CKM syndrome Stage 3. 95% CI, 95% confidence interval; LPA, light-intensity physical activity; MVPA, moderate-to-vigorous-intensity physical activity; CVD, cardiovascular disease; CKM, cardiovascular-kidney-metabolic.

| Level | physical activity  category | No. of cases/total  participants | Incidence rate per 1000 person-year |  | Hazard ratio (95% CI) |  |
| --- | --- | --- | --- | --- | --- | --- |
|  |  |  |  | Model 1 | Model 2 | Model 3 |
| LPA |  |  |  |  |  |  |
| Q1 | <1416min/week | 170/649 | 22.17 | Reference | Reference | Reference |
| Q2 | 1416-<1794min/week | 185/648 | 24.31 | 1.12 (0.91-1.38) | 1.14 (0.92-1.40) | 1.22(0.99-1.51) |
| Q3 | 1794-<2245min/week | 157/648 | 20.22 | 0.93 (0.75-1.16) | 0.94 (0.76-1.18) | 1.00(0.80-1.26) |
| Q4 | ≥2245min/week | 139/649 | 17.49 | 0.79 (0.63-0.99) | 0.81 (0.64-1.02) | 0.86(0.68-1.10) |
| MVPA |  |  |  |  |  |  |
| Q1 | <85min/week | 211/649 | 28.42 | Reference | Reference | Reference |
| Q2 | 85-<201min/week | 163/648 | 21.03 | 0.73 (0.60-0.90) | 0.74 (0.60-0.90) | 0.74(0.60-0.91) |
| Q3 | 201-<363min/week | 144/649 | 18.31 | 0.64 (0.52-0.79) | 0.65 (0.52-0.81) | 0.65(0.52-0.81) |
| Q4 | ≥363min/week | 133/648 | 16.72 | 0.60 (0.48-0.74) | 0.60 (0.48-0.76) | 0.600.48-0.75) |

Note: The quartile cut points for LPA and MVPA in this table are calculated based on the activity distribution of participants strictly within CKM Stage 3. Model 1 was adjusted for age, sex, ethnic, qualification, income and Townsend deprivation index; Model 2 was adjusted for age, sex, ethnic, qualification, income, Townsend deprivation index, smoking, drinking and sleep time; Model 3 was adjusted for age, sex, ethnic, qualification, income, Townsend deprivation index, smoking, drinking, sleep time, wear time and season. Model 3 with LPA terms was additionally adjusted for total MVPA minutes per week, whereas the Model 3 with MVPA terms was additionally adjusted for total LPA minutes per week.

## Table S6. Inflection point analysis of accelerometer-derived LPA and MVPA with CVD incidence in CKM syndrome stage 0-3. LPA, light-intensity physical activity; MVPA, moderate-to-vigorous-intensity physical activity; CVD, cardiovascular disease; CKM, cardiovascular-kidney-metabolic.

| Physical activity | CKM syndrome Stage | Slope | Estimated | Estimated Standard Error | t.value | upper CI | lower CI |
| --- | --- | --- | --- | --- | --- | --- | --- |
| LPA | Stage 0 | slope1 | -1.12E-04 | 6.53E-07 | -170.98 | -1.13E-04 | -1.10E-04 |
|  |  | slope2 | 5.82E-05 | 1.49E-06 | 39.15 | 5.52E-05 | 6.11E-05 |
|  | Stage 1 | slope1 | 8.78E-05 | 1.26E-05 | 6.9719 | 6.28E-05 | 1.13E-04 |
|  |  | slope2 | -7.90E-05 | 3.71E-06 | -21.291 | -8.64E-05 | -7.17E-05 |
|  | Stage 2 | slope1 | -3.25E-04 | 1.46E-06 | -223.37 | -3.28E-04 | -3.22E-04 |
|  |  | slope2 | -5.55E-05 | 9.29E-07 | -59.782 | -5.74E-05 | -5.37E-05 |
|  | Stage 3 | slope1 | 2.89E-04 | 1.49E-05 | 19.466 | 2.60E-04 | 3.19E-04 |
|  |  | slope2 | -1.67E-04 | 5.02E-06 | -33.311 | -1.77E-04 | -1.57E-04 |
|  | Stage 0-3 | slope1 | -1.82E-04 | 6.8038e-07 | -2.67E+02 | -1.83E-04 | -1.80E-04 |
|  |  | slope2 | -5.45E-05 | 1.0033e-06 | -5.44E+01 | -5.65E-05 | -5.25E-05 |
| MVPA | Stage 0 | slope1 | -1.40E-03 | 2.60E-05 | -53.835 | -1.45E-03 | -1.35E-03 |
|  |  | slope2 | -2.70E-04 | 3.81E-06 | -70.876 | -2.78E-04 | -2.63E-04 |
|  | Stage 1 | slope1 | -1.40E-03 | 2.60E-05 | -53.835 | -1.45E-03 | -1.35E-03 |
|  |  | slope2 | -2.70E-04 | 3.81E-06 | -70.876 | -2.78E-04 | -2.63E-04 |
|  | Stage 2 | slope1 | -2.83E-03 | 3.86E-05 | -73.319 | -2.91E-03 | -2.75E-03 |
|  |  | slope2 | -2.20E-04 | 2.49E-06 | -88.705 | -2.25E-04 | -2.16E-04 |
|  | Stage 3 | slope1 | -1.68E-03 | 3.31E-05 | -50.803 | -1.75E-03 | -1.62E-03 |
|  |  | slope2 | -3.73E-04 | 8.46E-06 | -44.036 | -3.89E-04 | -3.56E-04 |
|  | Stage 0-3 | slope1 | -2.49E-03 | 4.9186e-05 | -5.06E+01 | -2.59E-03 | -2.39E-03 |
|  |  | slope2 | -2.50E-04 | 4.4707e-06 | -5.60E+01 | -2.59E-04 | -2.42E-04 |

Note: The analysis is based on Model 3, which was adjusted for age, sex, ethnicity, qualification, income, Townsend deprivation index, smoking, drinking, sleep time, wear time, and season. Model 3 with LPA terms was additionally adjusted for total MVPA minutes per week, whereas Model 3 with MVPA terms was additionally adjusted for total LPA minutes per week.

## Table S7. The risk matrix for the joint association of accelerometer-derived LPA and MVPA with CVD incidence in CKM syndrome stage 0-3. LPA, light-intensity physical activity; MVPA, moderate-to-vigorous-intensity physical activity; CVD, cardiovascular disease; CKM, cardiovascular-kidney-metabolic.

| **LPA (min/week)** | **MVPA (min/week)** | **No. of cases/**  **Total participants** | **Hazard ratio (95% CI)** |
| --- | --- | --- | --- |
| <1637 | <112 | 956/6585 | 1.000 (1.000-1.000) |
| <1637 | 112-231 | 519/5083 | 0.721 (0.647-0.802) |
| <1637 | 231-401 | 460/5109 | 0.644 (0.576-0.721) |
| <1637 | 401-3000 | 410/5284 | 0.541 (0.481-0.608) |
| 1637-2069 | <112 | 599/5183 | 0.932 (0.841-1.033) |
| 1637-2069 | 112-231 | 461/5353 | 0.669 (0.599-0.749) |
| 1637-2069 | 231-401 | 430/5518 | 0.607 (0.541-0.681) |
| 1637-2069 | 401-3000 | 482/6010 | 0.581 (0.520-0.650) |
| 2069-2551 | <112 | 464/5028 | 0.775 (0.693-0.868) |
| 2069-2551 | 112-231 | 432/5621 | 0.641 (0.571-0.720) |
| 2069-2551 | 231-401 | 419/5772 | 0.592 (0.527-0.665) |
| 2069-2551 | 401-3000 | 370/5639 | 0.504 (0.446-0.569) |
| 2552-5917 | <112 | 460/5264 | 0.802 (0.715-0.899) |
| 2552-5917 | 112-231 | 409/6008 | 0.621 (0.551-0.699) |
| 2552-5917 | 231-401 | 332/5665 | 0.529 (0.466-0.601) |
| 2552-5917 | 401-3000 | 309/5127 | 0.516 (0.453-0.588) |

Note: The analysis is based on Model 3, which was adjusted for age, sex, ethnicity, qualification, income, Townsend deprivation index, smoking, drinking, sleep time, wear time, and season. Model 3 with LPA terms was additionally adjusted for total MVPA minutes per week, whereas Model 3 with MVPA terms was additionally adjusted for total LPA minutes per week. The marginal sum of participants in this joint risk matrix may slightly differ from the exact quartile counts in the main analysis (*Table 2*) because the absolute cut-off values for LPA and MVPA intervals were kept strictly consistent across analyses.

## Table S8. The risk matrix for the joint association of accelerometer-derived LPA and MVPA with CVD incidence in CKM syndrome stage 0. LPA, light-intensity physical activity; MVPA, moderate-to-vigorous-intensity physical activity; CVD, cardiovascular disease; CKM, cardiovascular-kidney-metabolic.

| **LPA (min/week)** | **MVPA (min/week)** | **No. of cases/**  **Total participants** | **Hazard ratio (95% CI)** |
| --- | --- | --- | --- |
| <1780 | <155 | 64/1104 | Reference |
| <1780 | 156-284 | 46/989 | 0.883 (0.603-1.292) |
| <1780 | 285-458 | 60/1061 | 1.003 (0.703-1.432) |
| <1780 | 458-2509 | 45/1179 | 0.611 (0.415-0.900) |
| 1780-2211 | <155 | 53/983 | 1.001 (0.693-1.446) |
| 1780-2211 | 156-284 | 53/1066 | 0.979 (0.678-1.414) |
| 1780-2211 | 285-458 | 48/1143 | 0.759 (0.520-1.106) |
| 1780-2211 | 458-2509 | 58/1143 | 0.813 (0.567-1.167) |
| 2212-2687 | <155 | 53/1070 | 0.915 (0.631-1.325) |
| 2212-2687 | 156-284 | 47/1086 | 0.844 (0.577-1.237) |
| 2212-2687 | 285-458 | 45/1082 | 0.765 (0.520-1.126) |
| 2212-2687 | 458-2509 | 31/1097 | 0.503 (0.326-0.777) |
| 2687-5533 | <155 | 57/1178 | 1.006 (0.696-1.454) |
| 2687-5533 | 156-284 | 48/1194 | 0.800 (0.545-1.175) |
| 2687-5533 | 285-458 | 41/1050 | 0.804 (0.539-1.201) |
| 2687-5533 | 458-2509 | 29/914 | 0.606 (0.387-0.949) |

Note: The analysis is based on Model 3, which was adjusted for age, sex, ethnicity, qualification, income, Townsend deprivation index, smoking, drinking, sleep time, wear time, and season. Model 3 with LPA terms was additionally adjusted for total MVPA minutes per week, whereas Model 3 with MVPA terms was additionally adjusted for total LPA minutes per week. The marginal sum of participants in this joint risk matrix may slightly differ from the exact quartile counts in *Table S2* because the absolute cut-off values for LPA and MVPA intervals were kept strictly consistent across analyses.

## Table S9. The risk matrix for the joint association of accelerometer-derived LPA and MVPA with CVD incidence in CKM syndrome stage 1. LPA, light-intensity physical activity; MVPA, moderate-to-vigorous-intensity physical activity; CVD, cardiovascular disease; CKM, cardiovascular-kidney-metabolic.

| **LPA (min/week)** | **MVPA (min/week)** | **No. of cases/**  **Total participants** | **Hazard ratio (95% CI)** |
| --- | --- | --- | --- |
| <1667 | <115 | 177/1676 | Reference |
| <1667 | 115-<233 | 108/1447 | 0.699 (0.549-0.889) |
| <1667 | 233-<405 | 105/1497 | 0.655 (0.513-0.836) |
| <1667 | ≥405 | 91/1565 | 0.507 (0.392-0.656) |
| 1667-<2095 | <115 | 147/1563 | 1.038 (0.832-1.294) |
| 1667-<2095 | 115-<233 | 113/1606 | 0.733 (0.578-0.930) |
| 1667-<2095 | 233-<405 | 124/1654 | 0.774 (0.614-0.976) |
| 1667-<2095 | ≥405 | 125/1777 | 0.616 (0.487-0.778) |
| 2095-<2568 | <115 | 111/1502 | 0.861 (0.676-1.096) |
| 2095-<2568 | 115-<233 | 114/1777 | 0.704 (0.554-0.893) |
| 2095-<2568 | 233-<405 | 92/1725 | 0.565 (0.438-0.729) |
| 2095-<2568 | ≥405 | 97/1804 | 0.516 (0.401-0.665) |
| ≥2568 | <115 | 120/1658 | 0.930 (0.733-1.182) |
| ≥2568 | 115-<233 | 88/1863 | 0.576 (0.443-0.748) |
| ≥2568 | 233-<405 | 84/1716 | 0.583 (0.447-0.759) |
| ≥2568 | ≥405 | 78/1561 | 0.550 (0.419-0.723) |

Note: The analysis is based on Model 3, which was adjusted for age, sex, ethnicity, qualification, income, Townsend deprivation index, smoking, drinking, sleep time, wear time, and season. Model 3 with LPA terms was additionally adjusted for total MVPA minutes per week, whereas Model 3 with MVPA terms was additionally adjusted for total LPA minutes per week. The marginal sum of participants in this joint risk matrix may slightly differ from the exact quartile counts in *Table S3* because the absolute cut-off values for LPA and MVPA intervals were kept strictly consistent across analyses.

## Table S10. The risk matrix for the joint association of accelerometer-derived LPA and MVPA with CVD incidence in CKM syndrome stage 2. 95% CI, 95% confidence interval; LPA, light-intensity physical activity; MVPA, moderate-to-vigorous-intensity physical activity; CVD, cardiovascular disease; CKM, cardiovascular-kidney-metabolic.

| **LPA (min/week)** | **MVPA (min/week)** | **No. of cases/**  **Total participants** | **Hazard ratio (95% CI)** |
| --- | --- | --- | --- |
| <1580 | <98 | 604/3953 | Reference |
| <1580 | 98-<211 | 324/2745 | 0.764 (0.667-0.876) |
| <1580 | 211-<374 | 261/2573 | 0.651 (0.562-0.754) |
| <1580 | ≥374 | 233/2470 | 0.611 (0.524-0.712) |
| 1580-<2008 | <98 | 365/2809 | 0.931 (0.817-1.062) |
| 1580-<2008 | 98-<211 | 272/2700 | 0.683 (0.591-0.788) |
| 1580-<2008 | 211-<374 | 214/2549 | 0.564 (0.482-0.660) |
| 1580-<2008 | ≥374 | 255/2628 | 0.630 (0.543-0.731) |
| 2008-<2493 | <98 | 278/2641 | 0.767 (0.664-0.886) |
| 2008-<2493 | 98-<211 | 225/2606 | 0.612 (0.524-0.714) |
| 2008-<2493 | 211-<374 | 239/2534 | 0.651 (0.560-0.757) |
| 2008-<2493 | ≥374 | 201/2222 | 0.589 (0.501-0.692) |
| ≥2493 | <98 | 272/2562 | 0.806 (0.696-0.933) |
| ≥2493 | 98-<211 | 228/2655 | 0.641 (0.548-0.748) |
| ≥2493 | 211-<374 | 179/2335 | 0.568 (0.479-0.672) |
| ≥2493 | ≥374 | 159/1943 | 0.574 (0.481-0.686) |

Note: The analysis is based on Model 3, which was adjusted for age, sex, ethnicity, qualification, income, Townsend deprivation index, smoking, drinking, sleep time, wear time, and season. Model 3 with LPA terms was additionally adjusted for total MVPA minutes per week, whereas Model 3 with MVPA terms was additionally adjusted for total LPA minutes per week. The marginal sum of participants in this joint risk matrix may slightly differ from the exact quartile counts in *Table S4* because the absolute cut-off values for LPA and MVPA intervals were kept strictly consistent across analyses.

## Table S11. The risk matrix for the joint association of accelerometer-derived LPA and MVPA with CVD incidence in CKM syndrome stage 3. 95% CI, 95% confidence interval; LPA, light-intensity physical activity; MVPA, moderate-to-vigorous-intensity physical activity; CVD, cardiovascular disease; CKM, cardiovascular-kidney-metabolic.

| **LPA (min/week)** | **MVPA (min/week)** | **No. of cases/**  **Total participants** | **Hazard ratio (95% CI)** |
| --- | --- | --- | --- |
| <1416 | <85 | 133/405 | Reference |
| <1416 | 85-<201 | 60/225 | 0.810 (0.596-1.102) |
| <1416 | 201-<363 | 42/194 | 0.630 (0.443-0.897) |
| <1416 | ≥363 | 44/201 | 0.636 (0.448-0.905) |
| 1416-<1794 | <85 | 51/181 | 0.865 (0.624-1.201) |
| 1416-<1794 | 85-<201 | 36/165 | 0.619 (0.424-0.904) |
| 1416-<1794 | 201-<363 | 48/174 | 0.847 (0.605-1.186) |
| 1416-<1794 | ≥363 | 30/163 | 0.539 (0.359-0.807) |
| 1794-<2245 | <85 | 45/149 | 0.927 (0.655-1.312) |
| 1794-<2245 | 85-<201 | 33/136 | 0.710 (0.481-1.048) |
| 1794-<2245 | 201-<363 | 23/130 | 0.507 (0.323-0.796) |
| 1794-<2245 | ≥363 | 24/117 | 0.602 (0.386-0.937) |
| ≥2245 | <85 | 22/93 | 0.665 (0.419-1.056) |
| ≥2245 | 85-<201 | 27/87 | 0.928 (0.607-1.419) |
| ≥2245 | 201-<363 | 18/98 | 0.523 (0.315-0.867) |
| ≥2245 | ≥363 | 15/76 | 0.594 (0.344-1.026) |

Note: The analysis is based on Model 3, which was adjusted for age, sex, ethnicity, qualification, income, Townsend deprivation index, smoking, drinking, sleep time, wear time, and season. Model 3 with LPA terms was additionally adjusted for total MVPA minutes per week, whereas Model 3 with MVPA terms was additionally adjusted for total LPA minutes per week. The marginal sum of participants in this joint risk matrix may slightly differ from the exact quartile counts in *Table S5* because the absolute cut-off values for LPA and MVPA intervals were kept strictly consistent across analyses.

## Table S12. Subgroup analysis of accelerometer-derived LPA and MVPA with CVD incidence in CKM syndrome stage 0 (highest versus lowest quarter of physical activity). 95% CI, 95% confidence interval; LPA, light-intensity physical activity; MVPA, moderate-to-vigorous-intensity physical activity; CVD, cardiovascular disease; CKM, cardiovascular-kidney-metabolic.

|  | LPA | | |  | MVPA | | |  |
| --- | --- | --- | --- | --- | --- | --- | --- | --- |
| Subgroup | No.of cases | Total participants | Hazard ratio  (95% CI) | P for interaction | No.of cases | Total participants | Hazard ratio (95% CI) | P for interaction |
| ***Age*** | | | | |  | | |  |
| >60 years | 82 | 1064 | 0.820 (0.605-1.111) | 0.159 | 80 | 1063 | 0.595 (0.440-0.806) | 0.703 |
| ≤60 years | 92 | 3271 | 1.205 (0.885-1.642) |  | 90 | 3270 | 0.666 (0.498-0.890) |  |
| ***Sex*** | | | | |  | | |  |
| female | 105 | 3135 | 0.857 (0.641-1.146) | 0.930 | 77 | 3134 | 0.673 (0.507-0.894) | 0.361 |
| male | 92 | 1200 | 0.848 (0.626-1.147) |  | 63 | 1201 | 0.665 (0.485-0.913) |  |
| ***Qualification*** | | | | |  | | |  |
| Not College or university degree | 92 | 2078 | 0.857 (0.644-1.141) | 0.593 | 86 | 2076 | 0.586 (0.441-0.781) | 0.579 |
| College or university degree | 79 | 2257 | 0.987 (0.712-1.368) |  | 78 | 2257 | 0.652 (0.479-0.887) |  |
| ***Income*** | | | | |  | | |  |
| Before tax ≤52,000 EUR | 113 | 2698 | 0.805 (0.624-1.038) | 0.024 | 114 | 2699 | 0.612 (0.476-0.787) | 0.242 |
| Before tax >52,000 EUR | 55 | 1637 | 1.335 (0.875-2.038) |  | 55 | 1633 | 0.842 (0.572-1.240) |  |
| ***Townsend deprivation index*** | | | | |  | | |  |
| >2.43 | 64 | 2106 | 0.712 (0.510-0.993) | 0.123 | 65 | 2101 | 0.641 (0.462-0.889) | 0.825 |
| ≤2.43 | 107 | 2230 | 1.148 (0.858-1.536) |  | 99 | 2230 | 0.676 (0.513-0.892) |  |
| ***Smoking*** | | | | |  | | |  |
| Current | 10 | 243 | 0.600 (0.267-1.345) | 0.293 | 9 | 243 | 0.340 (0.145-0.794) | 0.356 |
| Never | 98 | 2758 | 0.975 (0.727-1.307) |  | 99 | 2758 | 0.705 (0.535-0.931) |  |
| Previous | 67 | 1328 | 1.032 (0.721-1.477) |  | 57 | 1328 | 0.654 (0.460-0.930) |  |
| ***Drinking*** | | | | |  | | |  |
| No | 14 | 218 | 2.110 (0.771-5.772) | 0.076 | 8 | 218 | 0.705 (0.267-1.857) | 0.901 |
| Yes | 160 | 4118 | 0.883 (0.706-1.103) |  | 156 | 4116 | 0.640 (0.516-0.794) |  |

Note: The analysis is based on Model 3, which was adjusted for age, sex, ethnicity, qualification, income, Townsend deprivation index, smoking, drinking, sleep time, wear time, and season. Model 3 with LPA terms was additionally adjusted for total MVPA minutes per week, whereas Model 3 with MVPA terms was additionally adjusted for total LPA minutes per week.

## Table S13. Subgroup analysis of accelerometer-derived LPA and MVPA with CVD incidence in CKM syndrome stage 1 (highest versus lowest quarter of physical activity). 95% CI, 95% confidence interval; LPA, light-intensity physical activity; MVPA, moderate-to-vigorous-intensity physical activity; CVD, cardiovascular disease; CKM, cardiovascular-kidney-metabolic.

|  | LPA | | | | MVPA | | | |
| --- | --- | --- | --- | --- | --- | --- | --- | --- |
| Subgroup | No.of cases | Total participants | Hazard ratio (95% CI) | P for interaction | No.of cases | Total participants | Hazard ratio (95% CI) | P for interaction |
| ***Age*** | | | | | | | | |
| >60 years | 216 | 2148 | 0.980 (0.809-1.186) | 0.634 | 220 | 2148 | 0.578 (0.480-0.695) | 0.914 |
| ≤60 years | 153 | 4452 | 0.832 (0.669-1.035) |  | 184 | 4452 | 0.561 (0.459-0.684) |  |
| ***Sex*** | | | | | | | | |
| female | 165 | 4068 | 0.748 (0.606-0.925) | 0.005 | 117 | 4066 | 0.511 (0.409-0.637) | 0.001 |
| male | 240 | 2532 | 1.067 (0.884-1.289) |  | 203 | 2532 | 0.671 (0.562-0.803) |  |
| ***Qualification*** | | | | | | | | |
| Not College or university degree | 213 | 3753 | 0.830 (0.691-0.998) | 0.02 | 242 | 3749 | 0.653 (0.547-0.780) | 0.540 |
| College or university degree | 150 | 2843 | 1.077 (0.854-1.358) |  | 151 | 2844 | 0.539 (0.436-0.667) |  |
| ***Income*** | | | | | | | | |
| Before tax ≤52,000 EUR | 250 | 4408 | 0.862 (0.727-1.022) | 0.124 | 271 | 4406 | 0.554 (0.472-0.652) | 0.134 |
| Before tax >52,000 EUR | 102 | 2189 | 0.984 (0.744-1.301) |  | 119 | 2189 | 0.733 (0.565-0.950) |  |
| ***Townsend deprivation index*** | | | | | | | | |
| >2.43 | 186 | 3290 | 0.973 (0.793-1.193) | 0.199 | 171 | 3287 | 0.564 (0.461-0.689) | 0.015 |
| ≤2.43 | 172 | 3308 | 0.810 (0.660-0.995) |  | 217 | 3305 | 0.606 (0.503-0.731) |  |
| ***Smoking*** | | | | | | | | |
| Current | 23 | 373 | 0.618 (0.356-1.072) | 0.492 | 23 | 373 | 0.465 (0.273-0.793) | 0.764 |
| Never | 184 | 3860 | 0.950 (0.773-1.167) |  | 206 | 3857 | 0.606 (0.501-0.732) |  |
| Previous | 151 | 2347 | 0.895 (0.717-1.118) |  | 154 | 2347 | 0.590 (0.475-0.733) |  |
| ***Drinking*** | | | | | | | | |
| No | 18 | 331 | 1.114 (0.583-2.130) | 0.898 | 15 | 331 | 0.422 (0.226-0.789) | 0.005 |
| Yes | 341 | 6265 | 0.898 (0.774-1.043) |  | 370 | 6261 | 0.600 (0.521-0.690) |  |

Note: The analysis is based on Model 3, which was adjusted for age, sex, ethnicity, qualification, income, Townsend deprivation index, smoking, drinking, sleep time, wear time, and season. Model 3 with LPA terms was additionally adjusted for total MVPA minutes per week, whereas Model 3 with MVPA terms was additionally adjusted for total LPA minutes per week.

## Table S14. Subgroup analysis of accelerometer-derived LPA and MVPA with CVD incidence in CKM syndrome stage 2 (highest versus lowest quarter of physical activity). 95% CI, 95% confidence interval; LPA, light-intensity physical activity; MVPA, moderate-to-vigorous-intensity physical activity; CVD, cardiovascular disease; CKM, cardiovascular-kidney-metabolic.

Note: The analysis is based on Model 3, which was adjusted for age, sex, ethnicity, qualification, income, Townsend deprivation index, smoking, drinking, sleep time, wear time, and season. Model 3 with LPA terms was additionally adjusted for total MVPA minutes per week, whereas Model 3 with MVPA terms was additionally adjusted for total LPA minutes per week.

|  | LPA | | | | MVPA | | | |
| --- | --- | --- | --- | --- | --- | --- | --- | --- |
| Subgroup | No.of cases | Total participants | Hazard ratio (95% CI) | P for interaction | No.of cases | Total participants | Hazard ratio (95% CI) | P for interaction |
| ***Age*** | | | | | | | | |
| >60 years | 477 | 3859 | 0.754 (0.666-0.854) | 0.087 | 516 | 3860 | 0.604 (0.536-0.681) | 0.106 |
| ≤60 years | 456 | 6626 | 0.847 (0.745-0.964) |  | 464 | 6621 | 0.692 (0.609-0.788) |  |
| ***Sex*** | | | | | | | | |
| female | 382 | 5196 | 0.765 (0.665-0.881) | 0.144 | 278 | 5189 | 0.557 (0.482-0.645) | <0.001 |
| male | 606 | 5284 | 0.833 (0.744-0.932) |  | 543 | 5287 | 0.706 (0.632-0.788) |  |
| ***Qualification*** | | | | | | | | |
| Not College or university degree | 570 | 6241 | 0.807 (0.721-0.904) | 0.277 | 645 | 6238 | 0.667 (0.599-0.742) | 0.711 |
| College or university degree | 333 | 4242 | 0.819 (0.705-0.953) |  | 319 | 4243 | 0.662 (0.570-0.770) |  |
| ***Income*** | | | | | | | | |
| Before tax ≤52,000 EUR | 710 | 7460 | 0.812 (0.733-0.899) | 0.583 | 745 | 7458 | 0.646 (0.585-0.713) | 0.309 |
| Before tax >52,000 EUR | 177 | 3026 | 0.752 (0.615-0.918) |  | 225 | 3024 | 0.717 (0.596-0.864) |  |
| ***Townsend deprivation index*** | | | | | | | | |
| >2.43 | 491 | 5239 | 0.867 (0.765-0.982) | 0.108 | 468 | 5230 | 0.651 (0.575-0.737) | 0.717 |
| ≤2.43 | 432 | 5241 | 0.742 (0.652-0.846) |  | 479 | 5246 | 0.681 (0.601-0.772) |  |
| ***Smoking*** | | | | | | | | |
| Current | 54 | 722 | 0.579 (0.409-0.819) | 0.541 | 67 | 721 | 0.700 (0.501-0.978) | 0.089 |
| Never | 452 | 5807 | 0.786 (0.690-0.895) |  | 473 | 5805 | 0.691 (0.609-0.784) |  |
| Previous | 418 | 3925 | 0.875 (0.765-1.001) |  | 407 | 3920 | 0.630 (0.552-0.719) |  |
| ***Drinking*** | | | | | | | | |
| No | 71 | 644 | 0.758 (0.550-1.046) | 0.244 | 60 | 644 | 0.383 (0.277-0.530) | 0.120 |
| Yes | 860 | 9840 | 0.813 (0.741-0.893) |  | 894 | 9841 | 0.693 (0.633-0.760) |  |

## Table S15. Subgroup analysis of accelerometer-derived LPA and MVPA with CVD incidence in CKM syndrome stage 3 (highest versus lowest quarter of physical activity). 95% CI, 95% confidence interval; LPA, light-intensity physical activity; MVPA, moderate-to-vigorous-intensity physical activity; CVD, cardiovascular disease; CKM, cardiovascular-kidney-metabolic.

Note: The analysis is based on Model 3, which was adjusted for age, sex, ethnicity, qualification, income, Townsend deprivation index, smoking, drinking, sleep time, wear time, and season. Model 3 with LPA terms was additionally adjusted for total MVPA minutes per week, whereas Model 3 with MVPA terms was additionally adjusted for total LPA minutes per week.

|  | LPA | | | | MVPA | | | |
| --- | --- | --- | --- | --- | --- | --- | --- | --- |
| Subgroup | No.of cases | Total participants | Hazard ratio (95% CI) | P for interaction | No.of cases | Total participants | Hazard ratio (95% CI) | P for interaction |
| ***Age*** | | | | | | | | |
| >60 years | 124 | 568 | 0.846 (0.659-1.086) | 0.243 | 119 | 568 | 0.630 (0.495-0.800) | 0.282 |
| ≤60 years | 16 | 81 | 0.804 (0.401-1.611) |  | 14 | 81 | 0.445 (0.225-0.877) |  |
| ***Sex*** | | | | | | | | |
| female | 12 | 53 | 0.885 (0.408-1.920) | 0.391 | 6 | 53 | 0.349 (0.132-0.926) | 0.032 |
| male | 130 | 595 | 0.886 (0.692-1.135) |  | 121 | 596 | 0.630 (0.498-0.798) |  |
| ***Qualification*** | | | | | | | | |
| Not College or university degree | 93 | 431 | 0.885 (0.663-1.182) | 0.527 | 93 | 431 | 0.611 (0.465-0.802) | 0.781 |
| College or university degree | 48 | 217 | 0.758 (0.506-1.136) |  | 42 | 217 | 0.629 (0.423-0.936) |  |
| ***Income*** | | | | | | | | |
| Before tax ≤52,000 EUR | 114 | 546 | 0.832 (0.642-1.078) | 0.954 | 122 | 546 | 0.688 (0.541-0.875) | 0.29 |
| Before tax >52,000 EUR | 22 | 103 | 0.773 (0.426-1.402) |  | 15 | 103 | 0.304 (0.161-0.575) |  |
| ***Townsend deprivation index*** | | | | | | | | |
| >2.43 | 75 | 304 | 1.023 (0.731-1.432) | 0.1 | 70 | 304 | 0.640 (0.467-0.877) | 0.182 |
| ≤2.43 | 67 | 345 | 0.725 (0.520-1.011) |  | 62 | 344 | 0.538 (0.389-0.745) |  |
| ***Smoking*** | | | | | | | | |
| Current | 43 | 200 | 0.891 (0.582-1.365) | 0.782 | 39 | 200 | 0.681 (0.446-1.039) | 0.830 |
| Never | 41 | 200 | 0.807 (0.521-1.250) |  | 38 | 199 | 0.700 (0.456-1.075) |  |
| Previous | 61 | 248 | 1.020 (0.700-1.487) |  | 53 | 248 | 0.553 (0.388-0.788) |  |
| ***Drinking*** | | | | | | | | |
| No | 8 | 30 | 0.879 (0.334-2.316) | 0.428 | 10 | 29 | 0.906 (0.359-2.282) | 0 |
| Yes | 129 | 617 | 0.833 (0.652-1.064) |  | 124 | 617 | 0.607 (0.480-0.766) |  |

## Table S16. Sensitivity analysis of accelerometer-derived LPA and MVPA categories with CVD incidence in CKM syndrome stage 0-3, excluding events occurring within the first five years of follow-up. 95% CI, 95% confidence interval; LPA, light-intensity physical activity; MVPA, moderate-to-vigorous-intensity physical activity; CVD, cardiovascular disease; CKM, cardiovascular-kidney-metabolic.

| Level | physical activity  category | No. of cases/total  participants | Incidence rate per 1000 person-year |  | Hazard ratio (95% CI) |  |
| --- | --- | --- | --- | --- | --- | --- |
|  |  |  |  | Model 1 | Model 2 | Model 3 |
| LPA |  |  |  |  |  |  |
| Q1 | <1637min/week | 2111/22074 | 7.35 | Reference | Reference | Reference |
| Q2 | 1637-<2069min/week | 1756/22074 | 6.05 | 0.90(0.84-0.96) | 0.90(0.85-0.96) | 0.95(0.89-1.01) |
| Q3 | 2069-<2551min/week | 1493/22063 | 5.12 | 0.80(0.75-0.86) | 0.81(0.76-0.87) | 0.85(0.80-0.91) |
| Q4 | 2551-<5917min/week | 1372/22070 | 4.68 | 0.81(0.75-0.86) | 0.82(0.76-0.88) | 0.86(0.80-0.92) |
| MVPA |  |  |  |  |  |  |
| Q1 | <112min/week | 2228/22072 | 7.76 | Reference | Reference | Reference |
| Q2 | 112-<231min/week | 1629/22077 | 5.60 | 0.72(0.68-0.77) | 0.74(0.69-0.79) | 0.75(0.70-0.80) |
| Q3 | 231-<401min/week | 1470/22084 | 5.04 | 0.64(0.60-0.69) | 0.66(0.62-0.71) | 0.67(0.63-0.72) |
| Q4 | 401-<3000min/week | 1405/22048 | 4.80 | 0.58(0.54-0.62) | 0.60(0.56-0.64) | 0.60(0.56-0.64) |

Note: The quartile cut points for LPA and MVPA in this table are calculated based on the activity distribution of participants strictly within CKM Stage 0-3. Model 1 was adjusted for age, sex, ethnic, qualification, income and Townsend deprivation index; Model 2 was adjusted for age, sex, ethnic, qualification, income, Townsend deprivation index, smoking, drinking and sleep time; Model 3 was adjusted for age, sex, ethnic, qualification, income, Townsend deprivation index, smoking, drinking, sleep time, wear time and season. Model 3 with LPA terms was additionally adjusted for total MVPA minutes per week, whereas the Model 3 with MVPA terms was additionally adjusted for total LPA minutes per week.

## Table S17. Sensitivity analysis of accelerometer-derived LPA and MVPA categories with IHD incidence in CKM syndrome stages 0-3. 95% CI, 95% confidence interval; LPA, light-intensity physical activity; MVPA, moderate-to-vigorous-intensity physical activity; IHD, Ischemic Heart Disease; CKM, cardiovascular-kidney-metabolic.

| Level | physical activity  category | No. of cases/total  participants | Incidence rate per 1000 person-year |  | Hazard ratio (95% CI) |  |
| --- | --- | --- | --- | --- | --- | --- |
|  |  |  |  | Model 1 | Model 2 | Model 3 |
| LPA |  |  |  |  |  |  |
| Q1 | <1637min/week | 1662/22074 | 5.78 | Reference | Reference | Reference |
| Q2 | 1637-<2069min/week | 1371/22074 | 4.72 | 0.91(0.85-0.98) | 0.92(0.86-0.99) | 0.96(0.90-1.04) |
| Q3 | 2069-<2551min/week | 1152/22063 | 3.95 | 0.83(0.76-0.89) | 0.83(0.77-0.90) | 0.88(0.81-0.95) |
| Q4 | ≥2551min/week | 978/22070 | 3.33 | 0.78(0.72-0.85) | 0.79(0.73-0.86) | 0.83(0.76-0.90) |
| MVPA |  |  |  |  |  |  |
| Q1 | <112min/week | 1684/22072 | 5.87 | Reference | Reference | Reference |
| Q2 | 112-<231min/week | 1251/22077 | 4.30 | 0.72(0.67-0.78) | 0.74(0.69-0.80) | 0.75(0.69-0.80) |
| Q3 | 231-<401min/week | 1127/22084 | 3.86 | 0.63(0.59-0.68) | 0.65(0.60-0.70) | 0.66(0.61-0.71) |
| Q4 | ≥401min/week | 1101/22048 | 3.76 | 0.57(0.52-0.61) | 0.59(0.54-0.63) | 0.59(0.54-0.64) |

Note: The quartile cut points for LPA and MVPA in this table are calculated based on the activity distribution of participants strictly within CKM Stage 0-3. Model 1 was adjusted for age, sex, ethnic, qualification, income and Townsend deprivation index; Model 2 was adjusted for age, sex, ethnic, qualification, income, Townsend deprivation index, smoking, drinking and sleep time; Model 3 was adjusted for age, sex, ethnic, qualification, income, Townsend deprivation index, smoking, drinking, sleep time, wear time and season. Model 3 with LPA terms was additionally adjusted for total MVPA minutes per week, whereas the Model 3 with MVPA terms was additionally adjusted for total LPA minutes per week.

## Table S18. Sensitivity analysis of accelerometer-derived LPA and MVPA categories with stroke incidence in CKM syndrome stages 0-3. 95% CI, 95% confidence interval; LPA, light-intensity physical activity; MVPA, moderate-to-vigorous-intensity physical activity; CKM, cardiovascular-kidney-metabolic.

| Level | physical activity  category | No. of cases/total  participants | Incidence rate per 1000 person-year |  | Hazard ratio (95% CI) |  |
| --- | --- | --- | --- | --- | --- | --- |
|  |  |  |  | Model 1 | Model 2 | Model 3 |
| LPA |  |  |  |  |  |  |
| Q1 | <1637min/week | 429/22074 | 1.49 | Reference | Reference | Reference |
| Q2 | 1637-<2069min/week | 340/22074 | 1.17 | 0.83(0.72-0.96) | 0.86(0.74-0.99) | 0.88(0.76-1.02) |
| Q3 | 2069-<2551min/week | 309/22063 | 1.06 | 0.78(0.67-0.91) | 0.82(0.70-0.95) | 0.85(0.73-0.99) |
| Q4 | ≥2551min/week | 309/22070 | 1.05 | 0.85(0.73-0.99) | 0.91(0.78-1.07) | 0.94(0.80-1.10) |
| MVPA |  |  |  |  |  |  |
| Q1 | <112min/week | 432/22072 | 1.51 | Reference | Reference | Reference |
| Q2 | 112-<231min/week | 351/22077 | 1.21 | 0.82(0.71-0.94) | 0.85(0.73-0.97) | 0.86(0.74-0.99) |
| Q3 | 231-<401min/week | 311/22084 | 1.07 | 0.73(0.63-0.85) | 0.76(0.65-0.88) | 0.77(0.66-0.90) |
| Q4 | ≥401min/week | 293/22048 | 1.00 | 0.66(0.57-0.77) | 0.70(0.60-0.82) | 0.71(0.61-0.83) |

Note: The quartile cut points for LPA and MVPA in this table are calculated based on the activity distribution of participants strictly within CKM Stage 0-3. Model 1 was adjusted for age, sex, ethnic, qualification, income and Townsend deprivation index; Model 2 was adjusted for age, sex, ethnic, qualification, income, Townsend deprivation index, smoking, drinking and sleep time; Model 3 was adjusted for age, sex, ethnic, qualification, income, Townsend deprivation index, smoking, drinking, sleep time, wear time and season. Model 3 with LPA terms was additionally adjusted for total MVPA minutes per week, whereas the Model 3 with MVPA terms was additionally adjusted for total LPA minutes per week.

## Table S19. Sensitivity analysis of accelerometer-derived LPA and MVPA categories with HF incidence in CKM syndrome stages 0-3. 95% CI, 95% confidence interval; LPA, light-intensity physical activity; MVPA, moderate-to-vigorous-intensity physical activity; HF, heart failure; CKM, cardiovascular-kidney-metabolic.

| Level | physical activity  category | No. of cases/total  participants | Incidence rate per 1000 person-year |  | Hazard ratio (95% CI) |  |
| --- | --- | --- | --- | --- | --- | --- |
|  |  |  |  | Model 1 | Model 2 | Model 3 |
| LPA |  |  |  |  |  |  |
| Q1 | <1637min/week | 558/22074 | 1.94 | Reference | Reference | Reference |
| Q2 | 1637-<2069min/week | 408/22074 | 1.41 | 0.80(0.70-0.91) | 0.79(0.70-0.90) | 0.85(0.75-0.97) |
| Q3 | 2069-<2551min/week | 344/22063 | 1.18 | 0.70(0.61-0.81) | 0.70(0.61-0.80) | 0.76(0.66-0.88) |
| Q4 | ≥2551min/week | 316/22070 | 1.08 | 0.72(0.63-0.83) | 0.71(0.61-0.82) | 0.77(0.66-0.89) |
| MVPA |  |  |  |  |  |  |
| Q1 | <112min/week | 644/22072 | 2.24 | Reference | Reference | Reference |
| Q2 | 112-<231min/week | 382/22077 | 1.31 | 0.60(0.53-0.68) | 0.61(0.54-0.70) | 0.62(0.55-0.71) |
| Q3 | 231-<401min/week | 306/22084 | 1.05 | 0.49(0.42-0.56) | 0.50(0.43-0.57) | 0.51(0.44-0.58) |
| Q4 | ≥401min/week | 294/22048 | 1.00 | 0.45(0.39-0.51) | 0.46(0.40-0.53) | 0.46(0.40-0.53) |

Note: The quartile cut points for LPA and MVPA in this table are calculated based on the activity distribution of participants strictly within CKM Stage 0-3. Model 1 was adjusted for age, sex, ethnic, qualification, income and Townsend deprivation index; Model 2 was adjusted for age, sex, ethnic, qualification, income, Townsend deprivation index, smoking, drinking and sleep time; Model 3 was adjusted for age, sex, ethnic, qualification, income, Townsend deprivation index, smoking, drinking, sleep time, wear time and season. Model 3 with LPA terms was additionally adjusted for total MVPA minutes per week, whereas the Model 3 with MVPA terms was additionally adjusted for total LPA minutes per week.

## Figure S1. Cumulative risk of CVD incidence stratified by accelerometer-derived LPA and MVPA quartiles in CKM syndrome stage 0, 1, 2 and 3. LPA, light-intensity physical activity; MVPA, moderate-to-vigorous-intensity physical activity; CVD, cardiovascular disease; CKM, cardiovascular-kidney-metabolic. P-values were calculated using the log-rank test.

## Figure S2. Restricted Cubic Splines of accelerometer-derived LPA and MVPA with CVD incidence in CKM syndrome stage 0, 1, 2, 3 and 0-3. LPA, light-intensity physical activity; MVPA, moderate-to-vigorous-intensity physical activity; CVD, cardiovascular disease; CKM, cardiovascular-kidney-metabolic. The analysis is based on Model 3, which was adjusted for age, sex, ethnicity, qualification, income, Townsend deprivation index, smoking, drinking, sleep time, wear time, and season. Model 3 with LPA terms was additionally adjusted for total MVPA minutes per week, whereas Model 3 with MVPA terms was additionally adjusted for total LPA minutes per week.

## Figure S3. Inflection point analysis of accelerometer-derived LPA and MVPA with CVD incidence in CKM syndrome stage 0, 1, 2, 3 and 0-3. CVD, cardiovascular disease; CKM, cardiovascular-kidney-metabolic. The analysis is based on Model 3, which was adjusted for age, sex, ethnicity, qualification, income, Townsend deprivation index, smoking, drinking, sleep time, wear time, and season. Model 3 with LPA terms was additionally adjusted for total MVPA minutes per week, whereas Model 3 with MVPA terms was additionally adjusted for total LPA minutes per week.

## Figure S4. Population-attributable fractions of accelerometer-derived LPA and MVPA with CVD incidence in CKM syndrome stage 0, 1, 2 and 3. LPA, light-intensity physical activity; MVPA, moderate-to-vigorous-intensity physical activity; CVD, cardiovascular disease; CKM, cardiovascular-kidney-metabolic. Error bars represent the 95% confidence intervals. The analysis is based on Model 3, which was adjusted for age, sex, ethnicity, qualification, income, Townsend deprivation index, smoking, drinking, sleep time, wear time, and season. Model 3 with LPA terms was additionally adjusted for total MVPA minutes per week, whereas Model 3 with MVPA terms was additionally adjusted for total LPA minutes per week.

## Figure S5. The risk matrix for the joint association of accelerometer-derived LPA and MVPA with CVD incidence in CKM syndrome stage 0, 1, 2 and 3. LPA, light-intensity physical activity; MVPA, moderate-to-vigorous-intensity physical activity; CVD, cardiovascular disease; CKM, cardiovascular-kidney-metabolic. Numbers in the matrix represent the percentage of risk reduction (%) compared with the reference group (the matrix marked '0', representing the lowest quartiles of both LPA and MVPA). The analysis is based on Model 3, which was adjusted for age, sex, ethnicity, qualification, income, Townsend deprivation index, smoking, drinking, sleep time, wear time, and season. Model 3 with LPA terms was additionally adjusted for total MVPA minutes per week, whereas Model 3 with MVPA terms was additionally adjusted for total LPA minutes per week.
